# A Systematic Review of Mobile Phone Data in Crime Applications: A Coherent Taxonomy Based on Data Types and Analysis Perspectives, Challenges, and Future Research Directions

**DOI:** 10.3390/s23094350

**Published:** 2023-04-28

**Authors:** Mohammed Okmi, Lip Yee Por, Tan Fong Ang, Ward Al-Hussein, Chin Soon Ku

**Affiliations:** 1 Faculty of Computer Science and Information Technology, Universiti Malaya, Kuala Lumpur 50603, Malaysiawva180034@siswa.um.edu.my (W.A.-H.); 2 Department of Information Technology and Security, Jazan University, Jazan 45142, Saudi Arabia; 3 Department of Computer Science, Universiti Tunku Abdul Rahman, Kampar 31900, Malaysia

**Keywords:** mobile phone data, call detail records (CDRs), urban human mobility patterns, human communication behavior, urban dynamics, criminal networks, social networks, urban crime prediction, urban sensing, systematic literature review

## Abstract

Digital technologies have recently become more advanced, allowing for the development of social networking sites and applications. Despite these advancements, phone calls and text messages still make up the largest proportion of mobile data usage. It is possible to study human communication behaviors and mobility patterns using the useful information that mobile phone data provide. Specifically, the digital traces left by the large number of mobile devices provide important information that facilitates a deeper understanding of human behavior and mobility configurations for researchers in various fields, such as criminology, urban sensing, transportation planning, and healthcare. Mobile phone data record significant spatiotemporal (i.e., geospatial and time-related data) and communication (i.e., call) information. These can be used to achieve different research objectives and form the basis of various practical applications, including human mobility models based on spatiotemporal interactions, real-time identification of criminal activities, inference of friendship interactions, and density distribution estimation. The present research primarily reviews studies that have employed mobile phone data to investigate, assess, and predict human communication and mobility patterns in the context of crime prevention. These investigations have sought, for example, to detect suspicious activities, identify criminal networks, and predict crime, as well as understand human communication and mobility patterns in urban sensing applications. To achieve this, a systematic literature review was conducted on crime research studies that were published between 2014 and 2022 and listed in eight electronic databases. In this review, we evaluated the most advanced methods and techniques used in recent criminology applications based on mobile phone data and the benefits of using this information to predict crime and detect suspected criminals. The results of this literature review contribute to improving the existing understanding of where and how populations live and socialize and how to classify individuals based on their mobility patterns. The results show extraordinary growth in studies that utilized mobile phone data to study human mobility and movement patterns compared to studies that used the data to infer communication behaviors. This observation can be attributed to privacy concerns related to acquiring call detail records (CDRs). Additionally, most of the studies used census and survey data for data validation. The results show that social network analysis tools and techniques have been widely employed to detect criminal networks and urban communities. In addition, correlation analysis has been used to investigate spatial–temporal patterns of crime, and ambient population measures have a significant impact on crime rates.

## 1. Introduction

Even though digital technologies have become more advanced in recent times and allowed for the development of social networking sites, computer software applications, and emails, research findings have shown that phone calls and text messages still represent the greatest proportions of mobile data usage. Statista, a German company specializing in market and consumer data, estimated that the global number of mobile subscriptions would exceed 8 billion as of 2020. Whenever mobile phone users initiate an activity (e.g., calling, texting, and connecting to the Internet), this action is recorded by the mobile network operator. The information saved includes such details as each call’s duration, timestamp, and location at which the interaction started. When considering the aforementioned points along with the mobile subscription figure, it could be concluded that tremendous amounts of mobile phone data are generated every day. Maintaining such rich data, which comprise the details of individuals’ behaviors and activities, is advantageous in the sense that human communication behavior and mobility patterns can be studied at a low cost. The accessibility of this data has been reflected in various studies and disciplines over the years in terms of the ubiquitous use of mobile phone data [1]. For instance, publications have focused on urban sensing, safety, health, emergencies, transportation planning, and criminology.

Mobile phone data are log files collected from the users by mobile network operators during the service provision process. They contain all of the interactions that the user has initiated with the network, whether actively (e.g., when making a phone call, sending a text message, or accessing the internet) or passively (e.g., when switching the phone on or off, receiving a signal from the mobile network, or changing the type of connection). They also contain the details of each of these interactions, such as the phone numbers of the caller and receiver, the timestamp, and the duration and location of the interaction (i.e., the cell tower ID). Every telecommunications service provider (TSP) records users’ interactions with the cellular network whenever they engage in an activity on their mobile devices; here, the data are recorded in the service provider’s database.

Mobile phone data have proven to be the most prominent form of data, helping us understand the microscopic details of social networks, human mobility, and human behavioral patterns [1]. For instance, they have enabled us to understand how members of a target population (i.e., users) change their communication (e.g., calling) behavior and mobility patterns following an emergency event, such as a terrorist attack [2]. Unsurprisingly, mobile phone data have become a topic of discussion in various studies and the centerpiece of many real-world applications [3,4]. In such contexts, they are used to infer social ties and interactions among individuals [5], estimate daily population dynamics [6], map tourist travel behaviors [7,8], identify suspects [9], detect criminal networks based on communication behaviors [10,11], detect criminals based on mobility patterns [12], predict crimes and criminal behaviors [13,14], understand human mobility patterns in urban environments [15], and estimate human mobility and behavior under emergency events, such as natural disasters [16], migration streams [17], reprisals of organized criminals or militia, and the spread of infectious diseases.

When considering the applications described above, it becomes apparent that mobile phone data are among the most reliable sources of information that could help sense and record human activities. Moreover, they have a great potential for being used to reveal many aspects of human mobility patterns and communication behaviors. Therefore, using these data can help us to accurately and effectively predict and understand individual friendship relationships, criminal relationships, social ties, and interactions based on calling behaviors, as well as humans’ way of living, which has always been inextricably linked [18] with movement patterns.

In the last decade, mobile phone data have been used as sensors for detecting human mobility and communication behaviors. Due to the wide use of smartphones and the fast growth of telecommunication networks, a large quantity of data on how people move and behave across space and time has been recorded. The digital traces left by smartphones provide valuable, real-time information about various human activities.

For example, mobile phone data have been used to indicate the presence or absence of humans during certain times and at specific locations [19]. Thus, in criminal investigations, location-based mobile phone data could be used to indicate the presence of suspects in an area at a certain time where a crime has taken place, to monitor the spatial and temporal fluctuations of a population’s activities in a given area, to estimate the mobility flow of visitors, and to infer land use (i.e., commercial, industrial, residential, and educational) based on the total call volume or number of calls managed by a given cell tower over a given period of time [4,20]. Because these measures and assessments are extracted from mobile data, the data have become a topic of academic discussion and the centerpiece of many real-world applications.

For example, Refs. [21,22,23] depicted human mobility patterns from mobile phone data by extracting spatiotemporal features in the form of timestamps and cell tower IDs. These features were used to estimate or count the number of times a mobile phone device communicated with a given cell tower. These parameters and measurements aided in the investigation of the relationship between human mobility patterns and crime patterns.

Similarly, spatiotemporal features, such as cell tower IDs, timestamps, and call logs, have been extracted to depict other aspects of human activities, such as identifying residential and working activity to evaluate adherence to NPI policies, such as stay-at-home regulations or recommendations [24,25,26]; to estimate migration flow [18]; and to calculate the number of trips made between an origin (e.g., home) and destination (workplace) [27]. These measurements were calculated based on the definition of home and work locations, where home is indicated as the most frequently used or contacted cell tower during nighttime hours (7 p.m. to 7 a.m.) and work is indicated by the most frequently used cell tower during the day.

Another example of a practical application of human activity characteristics that can be extracted from mobile phone data is the detection of criminal social interactions. For example, social networks can be created among individuals making or receiving calls or messages who are classified as actors (nodes) within the network; each link between actors is represented by the type of communication (call or message). Some studies [28,29] have diagrammed criminal networks by analyzing criminal communication (calling) behaviors, such as call frequency, maximum and minimum numbers of incoming or outgoing calls and messages, and temporal changes in mobile phone call patterns. This process, wherein specific social groups are identified along with their internal structures and communities, is referred to as social network analysis (SNA). SNA can be harnessed to determine the relationships and interactions between criminals by reconstructing the communication relationships that are obtained from mobile phone data as a network, where a node represents a criminal and an edge represents a communication (i.e., a phone call or a message). This method of analysis has been widely adopted in mobile phone data studies since it can help criminal investigators determine who belongs to a criminal organization, who heads it, and the relationships that exist within it. Using this approach in the study of criminal networks allows criminal investigators and experts to understand a network’s hierarchy, its key leader, and subordinate leaders, and label the various levels of the criminal organization.

Here, we review existing studies that utilize mobile phone data with a particular focus on detecting and predicting criminal behaviors from people- and place-centric perspectives [13]. This includes studies that employ data on the prediction of crimes and criminal activities, the identification and detection of suspects and criminals, and other studies related to criminological research, such as exploring the relationship between human mobility patterns and crime patterns. We also shed light on the methods that employ mobile phone data to understand the dynamics of human behavior and mobility in urban sensing.

Although studies [1,3] made impressive contributions by exploring the applications of mobile phone data in social networking and urban sensing, knowledge about the use of mobile phone data in criminology research is lacking. Thus, a systematic review is needed to fill this gap by investigating the current state of mobile phone data use and applications in criminology research. Such an investigation would help to offer an overview of current approaches used to fight crime, prevent criminal activities, and detect criminal organizations. In addition, investigating these approaches can generate significant information about the tools and methods previously used to analyze mobile phone data, as well as provide a broader understanding of people’s actions and activities in the areas in which they live and socialize and categorize individuals according to their mobility patterns so that the authorities can determine population flows in these zones before and during crimes. Thus, this study was motivated by a desire to enable researchers to create effective methods for extracting useful information from mobile phone data. These well-designed methodologies will enhance the process of identifying suspects and predicting crimes and provide a more complete picture of the dynamics of criminal behaviors from a people- and place-centric perspective.

Thus, this review aims to examine and explore the applications derived from human behavioral patterns extracted from mobile phone data in criminology research and evaluate the characteristics of multiple analysis perspectives (mobility patterns, communication behaviors, and social interactions) that have been derived from mobile phone data to depict aspects of human behavior and activity. The review also focuses on analyzing and explaining the choice of human features and characteristics, such as spatiotemporal and call features, that have been extracted to model human mobility and communication patterns in the context of criminology.

### 1.1. Survey Analysis

Limited types of reviews and survey articles related to mobile phone data have summarized applications in the mobile phone data domain. Notably, Refs. [1,3,4] presented comprehensive surveys about different applications of mobile phone data. Blondel et al. [1] reviewed social network applications that can be derived from mobile phone data in various disciplines and domains, such as social relationships, urban sensing, epidemics, public transportation, data protection, and criminology, with a major focus on studies that construct social networks according to communications behavior (calling information). Calabrese et al. [3] presented a comprehensive review of mobile phone data applications in the urban sensing domain by discussing the different types of mobile phone datasets and processing techniques that have been created in this domain. Okmi et al. [4] presented surveys about different methods, characteristics, and features used for assessing and predicting human behaviors in various domains such as urban sensing, criminology, transportation, and health. Bhattacharya and Kaski [30] reviewed the human social network application, one of the social network applications in the mobile phone data domain. Ghahramani et al. [31] reviewed another survey paper about mobile phone data in the urban sensing domain. The authors presented a survey of the techniques and methods that have been used with mobile phone data in urban sensing applications, such as urban planning and public safety, by discussing the strengths and weaknesses of various approaches and comparing their advantages and disadvantages with those of other mobility datasets that capture people’s mobility patterns, including GPS, handover records, and location data.

Nevertheless, multiple analytical perspectives on mobile phone data that consider human communication behavior, social networks, and mobility patterns at various levels of mobile phone data (i.e., individual, aggregated, and cell tower data) have yet to be fully investigated. Studies of urban sensing domains are typically based on the use of mobile phone data that have been aggregated at the cell tower level, which provides only spatiotemporal information. Therefore, studies focusing on urban sensing domains have mostly discussed the analytical perspective of human mobility analysis patterns. Although [1] sheds light on various mobile phone data applications derived from different types of mobile phone data (i.e., individual, aggregated, and cell tower data), crime applications in mobile phone data have not been fully reviewed or discussed. Furthermore, new crime applications in mobile phone data have not been explored or investigated since that review.

**Figure 1:**
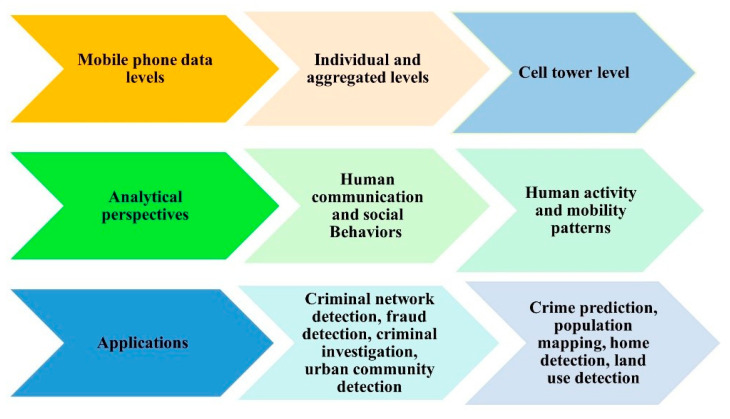
Multiple analytical perspectives and applications based on mobile phone data at various levels capture aspects of human behaviors.

This study differs from the previous survey framework in the sense that it examines and investigates various processing techniques and analytical perspectives that have been built based on mobile phone data at various levels to capture many aspects of human behaviors. These analytical perspectives, such as human mobility patterns, communication behaviors, social interactions, and mobile phone usage activities, have been built on multiple spatiotemporal and call characteristics extracted from mobile phone data. Even though Blondel et al. [1] aimed to review social network applications built on analyzing human social interactions that can be derived from mobile phone data and Calabrese et al. [3] presented a survey of urban sensing applications that are built on analyzing mobility patterns, knowledge about the use of mobile phone data in crime applications is still lacking. Thus, this study is the first to review the research focused on human mobility patterns, social interactions, and communication behaviors in crime applications and urban sensing applications. Figure 1 illustrates the multiple analytical perspectives and applications that this study has investigated and evaluated.

The primary contribution of this systematic literature review (SLR) is to provide a comprehensive overview of the applications of mobile phone data in crime-control research. As a comprehensive SLR on this topic is lacking, the present study represents the first attempt to carry out a critical analysis of this topic. To achieve this goal, a thorough search of eight top scientific databases (i.e., the Association for Computing Machinery Digital Library, Institute of Electrical and Electronics Engineers Xplore, Multidisciplinary Digital Publishing Institute, Sage, Science Direct, Scopus, SpringerLink, and Web of Science) was performed, and 107 primary studies that met the study’s scope and criteria were retrieved. This study involved four steps. The first was to extensively and systematically review the current state of mobile phone data use in crime applications, especially in those involving the identification and detection of criminals and the prediction of crimes. The second step was to investigate empirical research using mobile phone data to predict human behavior and mobility patterns in urban sensing applications. The third step involved providing a taxonomy for the final dataset of articles based on the scientific approach used and the research questions answered. The last step was to point out the potential challenges faced by this body of literature’s state-of-the-art techniques and to provide potential directions for future research.

### 1.2. Mobile Phone Data Types (Levels)

Generally, the mobile phone data record the users’ interactions on a mobile network and include details such as the IDs of the caller and the callee, the duration and timestamp of the interaction, and the location of the parties involved in the interaction (as determined by the cell tower ID). However, the data can be further divided into two types: a type that records the details of an interaction between a mobile device and the network, known as event-driven mobile phone data, and another type based on the cell tower location updates of mobile phones, known as network-driven mobile phone data (see Okmi et al. [4], Section 3, for more details about mobile phone data types).

The structure of the paper is organized as follows: In Section 2, we present the research methodology. In Section 3, we present the results of the SLR. In Section 4, the study taxonomy is presented. Section 5 addresses the research questions and discusses recent advances in detection methods. Section 6 discusses privacy concerns, investment behavior, and challenges. Section 7 defines the current problem and proposes a system model. Section 8 offers recommendations for future research and concludes the review.

## 2. Methodology

**Figure 2:**
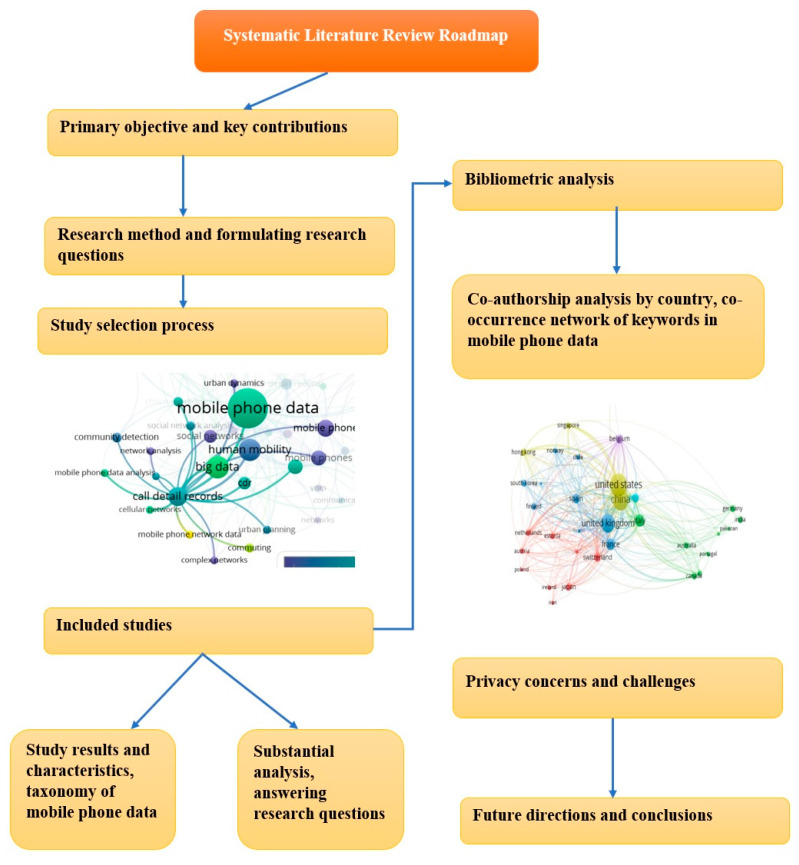
A systematic literature review roadmap that details the phases and stages investigated in this review will provide a more complete picture of the current state of research methods and techniques using mobile phone data.

This section outlines the research methodology used for the study. A systematic literature review was conducted by adopting Kitchenham’s guidelines [32] for search processes, inclusion and exclusion criteria, and data extraction. This study follows the reporting guidelines of “PRISMA” (“*Preferred Reporting Items for Systematic Reviews*”), which consist of a 27-item checklist and a 4-phase flow diagram for the selection of papers. The PRISMA statement by Liberati et al. [33] was used for the study selection process. This study also performed a bibliometric analysis along with the SLR to provide more thorough insights into the topic. Figure 2 shows the roadmap of the SLR, which clarified the planning of the review regarding the following points: the formulation of research questions, the study selection process, eligibility criteria (inclusion and exclusion criteria), bibliometrics and data extraction and synthesis strategies, study taxonomies, research questions, and future work. The systematic literature review road map begins with defining the main contributions and objectives of the review to allow the formulation of the research questions needed to achieve the study objectives. To answer these questions, a systematic review and bibliometric analysis were conducted to provide a thorough analysis of the topic. In the next stage, the studies were summarized, and a taxonomy based on the scientific approach was produced. This taxonomy helped to answer the research questions while establishing the current state of the research trends and applications of mobile phone data. In the final step of the road map, study limitations and future work were discussed.

### 2.1. Research Questions, Explanations, and Motivations

The comprehensive, systematic literature review presented in this research will focus on studies that have explored the use of mobile phone data to detect suspicious movements, crimes, and suspects; to predict human behaviors; and to understand human communication behavior. Two primary research questions have been developed for the present work, which are designed to determine the current status of mobile phone data and to investigate the different characteristics of studies that have employed mobile phone data in a variety of fields. As far as we are aware, there are no previous studies that have reviewed advancements in the field of mobile phone data using the specific inclusion criteria of the present research. This is a significant reason for which we wish to carry out this review. Below, we formulate two research questions with their explanations, as presented in Table 1 of this study.

1. RQ1: What are the current state-of-the-art methods and techniques regarding the use of mobile phone data in crime applications, especially in identifying suspects and predicting crimes?
2. RQ2: How can identifying empirical mobile phone data studies to predict human behavior and mobility patterns contribute to a clearer understanding of the dynamics of criminal behavior contexts from a people- and place-centric perspective?

### 2.2. Study Selection

**Figure 3:**
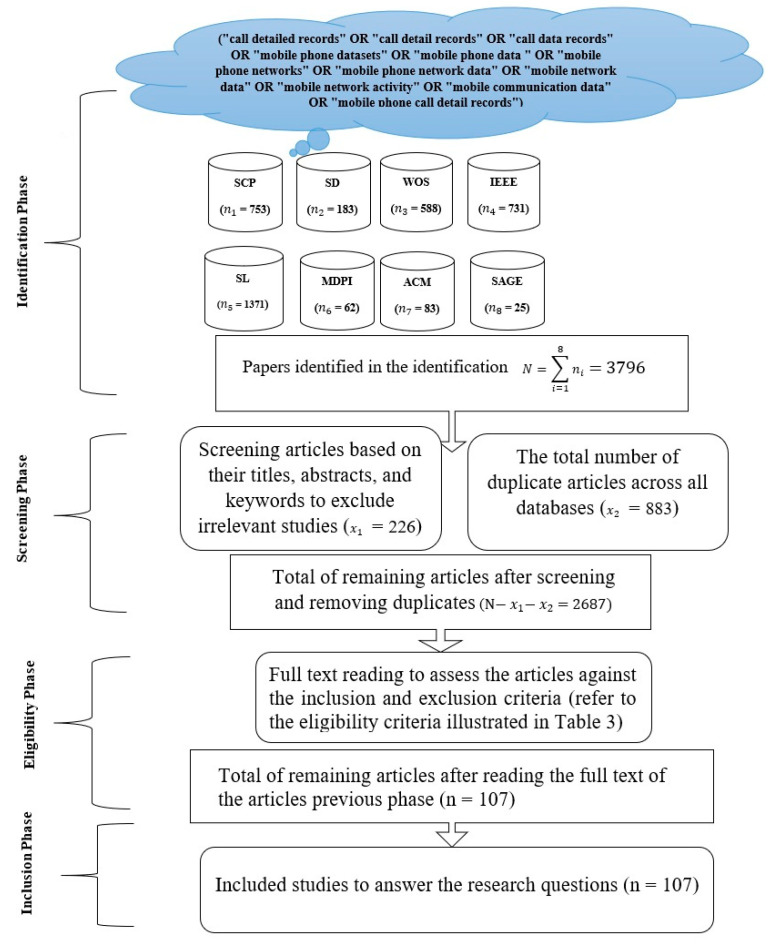
The four-phase flow diagram for the selection of papers.

The flow diagram for selecting candidate articles consists of the following phases: identification, screening, eligibility, and inclusion. Figure 3 shows the PRISMA flow diagram for the study selection processes.

The first phase comprised the process of identifying the most relevant research articles from reliable database sources by running the following search query (“call detailed records” OR “call detail records” OR “call data records” OR “mobile phone datasets” OR “mobile phone data” OR “mobile phone networks” OR “mobile phone network data” OR “mobile network data” OR “mobile network activity” OR “mobile communication data” OR “mobile phone call detail records”) under the “Search Within Title”, “Abstract”, and/or “Keywords” filters. The search query parameters were adjusted appropriately to account for the default configurations of the databases. A notable case is the AND operator, which is implemented by default between the Search Within terms. This makes it difficult to combine two search terms by using the OR operator against the Title, Abstract, and Keywords filters. For instance, the searches within the SAGE and MDPI databases were run against the Abstract filter because this yielded more results (i.e., publications) as compared to the Title and Keywords filters. Accordingly, the results obtained from the Abstract filter were ensured to be inclusive of the results produced by both the Title and Keywords filters. To ensure a comprehensive search of articles, the search query was run on the following eight database sources: ACM Digital Library, IEEE Xplore, MDPI, SAGE, Science Direct, Scopus, SpringerLink, and Web of Science. These databases offer highly advanced search options that allow the researchers to fine-tune their queries, in addition to their ability to produce accurate citation data, remove duplicated results, and exclude certain materials such as patents and gray literature. In contrast to the aforementioned databases, Google Scholar (GS) was excluded from the present study due to its limited search functionality, inaccurate reporting of metadata, and inability to remove duplicated results.

**Figure 4:**
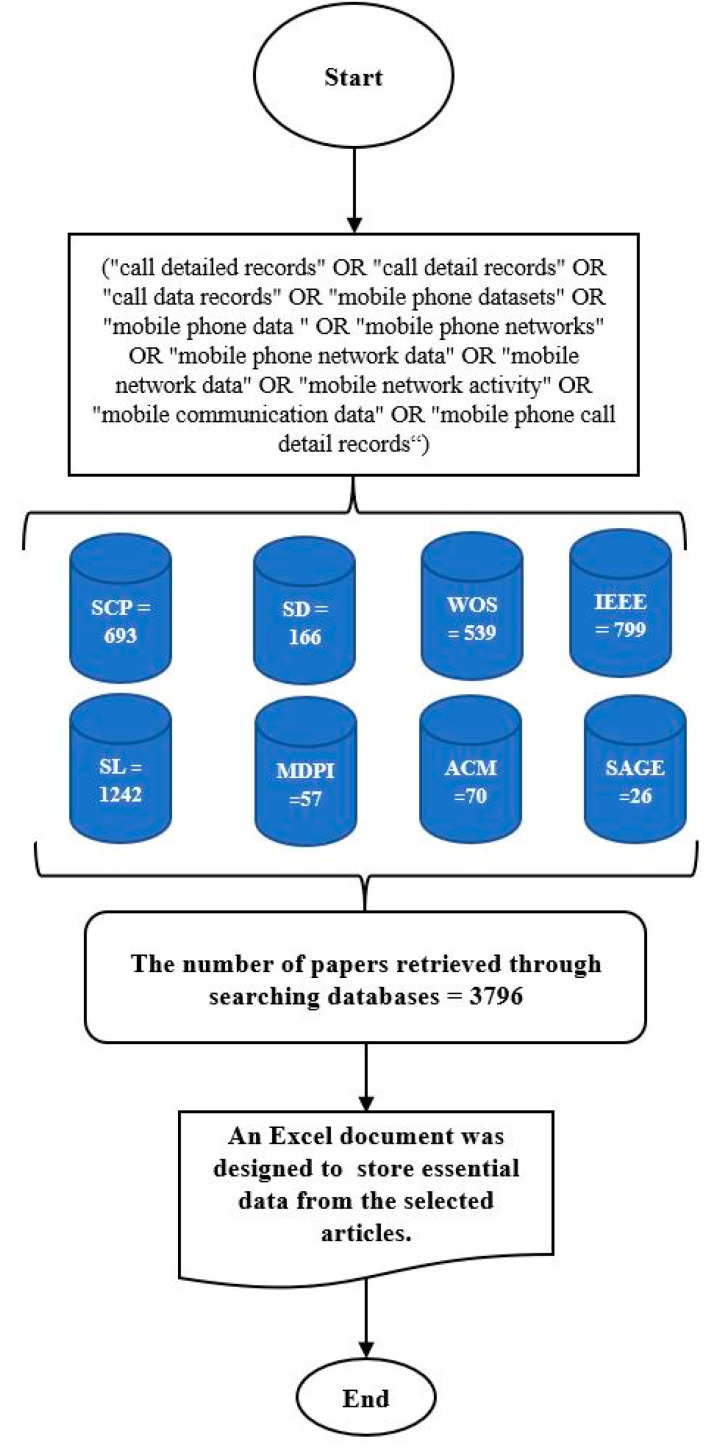
Flow chart for retrieving relevant articles through the search of databases.

The performed search yielded a total of 3796 publications based on the given criteria. The obtained data were imported into a Microsoft Excel spreadsheet and EndNote and later ordered by relevance in preparation for the subsequent phases. Figure 4 illustrates a flow chart for retrieving relevant studies through the search of databases.

The parameters that were used to search the database sources in the identification phase are presented in Table 2. These parameters facilitated the subsequent phases by setting the content language to English and the publication time to the period from 2014 through 2022. This ensured that articles written in languages other than English or before 2014 were omitted from the search results. It is noteworthy that, for the ScienceDirect database, the search query was iterated twice since the database only allows up to 8 OR operators to be included at once. Accordingly, the search was first performed with 8 keywords, then again with the remaining keywords. Subsequently, the results from each search iteration were merged together into one list.

The second phase was the screening phase, which incorporated the process of removing duplicates from the obtained list of publications across all databases, followed by a manual screening to exclude irrelevant articles based on their titles, abstracts, and keywords. This step is crucial because many articles may fall under the given search query but are published in irrelevant fields. This phase yielded a total of 2687 publications after removing duplication and irrelevant articles.

The third phase was the eligibility phase, which involved reading the full text of the articles selected from the previous phase. This phase assessed the articles against the inclusion criteria to determine their eligibility (refer to Table 3). Therefore, the total number of articles remaining after reading the full text is N = 107.

Finally, in the last phase, the articles chosen from the third phase were used to answer the research questions of the present study. To avoid bias, all of the phases were reviewed and performed by one author, and then a test–retest analysis was conducted by the second author to assess reliability.

### 2.3. Data Extraction and Synthesis Strategy

This section is essential for any SLR to aid in designing the data extraction form for the study results, and it is needed to help answer the research question. For this purpose, an Excel spreadsheet was created to store essential data from the selected articles. The data extraction form includes four parameters. The following data points were manually extracted:

DE1) The title of the article, the authors, the publishing journal, and other publication details.

DE2) Information related to mobile phone data types and their characteristics.

DE3) Information related to the mobile phone data domain and its applications; study area.

DE4) Information related to methods and techniques used in the mobile phone data domain.

The data synthesis was performed to accumulate and summarize the results of the included primary studies as well as to extract quantitative and qualitative data from the latter in forms that can be represented by tables, pie charts, bar and clustered bar charts, and scatter charts. VOSviewer software was used to obtain a visual representation of the data.

## 3. Results

This section presents a summary of the results obtained from the study selection process and includes details about the search results, the distribution of mobile phone data types, and a visualization of the co-occurrence of keywords. The distribution of publication type, publisher’s locations, most cited publications, distribution of analysis perspectives, and publication years are also provided.

### 3.1. Search Results

A total of 3796 studies were initially identified from the eight databases in the identification phase. In total, 2687 studies were subsequently excluded through the screening phase based on the filtering of the titles, abstracts, and keywords, which resulted in greatly decreasing the number of papers and removing duplicate papers obtained across all databases. Then, the results were further refined according to the eligibility criteria, and 2584 were removed based on the exclusion criteria. Eventually, 107 studies were included as the final set of selected articles in this review. Figure 3 depicts the four-phase flow diagram for the selection of papers.

### 3.2. Publications Years

**Figure 5:**
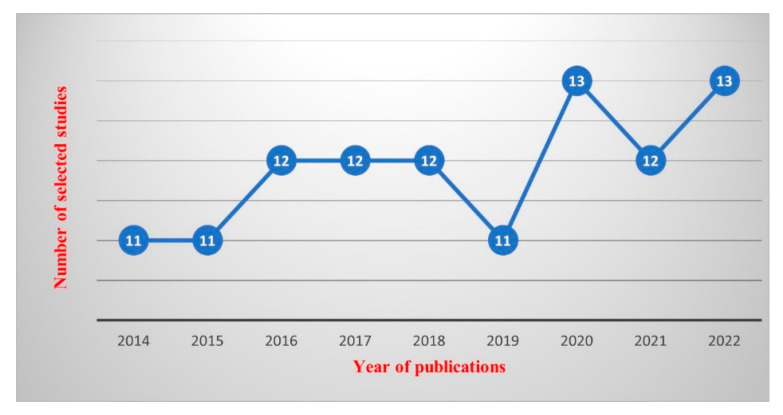
The distribution of selected articles by year of publication.

Figure 5 shows the publication years of selected studies as being between 2014 and 2022. It can be seen clearly that the research on mobile phone data has a steady indication of publications.

### 3.3. Publication Type

**Figure 6:**
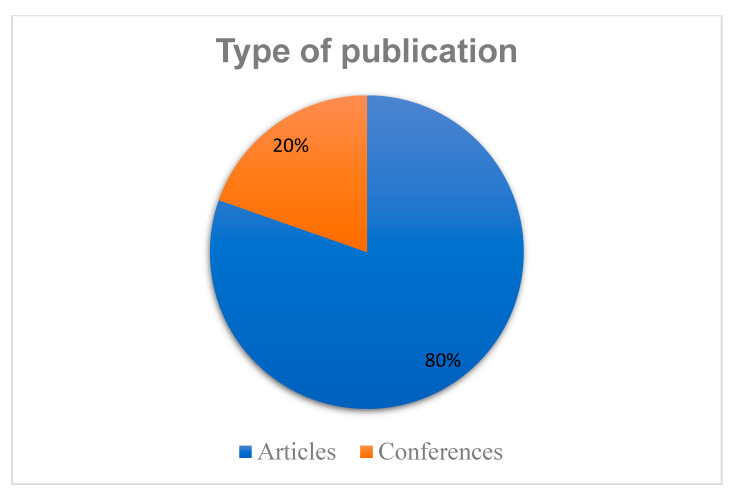
The distribution of publication types.

Figure 6 illustrates the distribution of publication types in mobile phone data. Out of the 107 primary studies selected, we observed that 86 (80%) appeared in articles and 21 (20%) were published in conferences. These statistics demonstrate that articles are the most active publication in the mobile phone data domain.

### 3.4. Mobile Phone Data Levels

**Figure 7:**
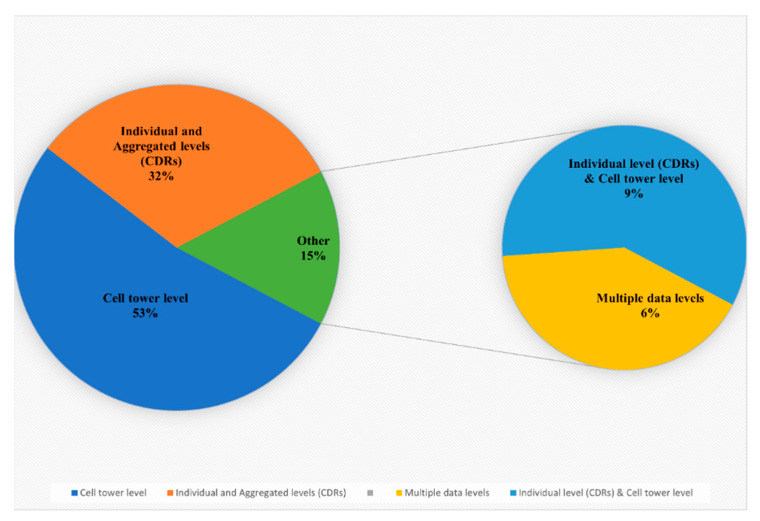
Distribution of mobile phone data types.

Figure 7 illustrates the distribution of all mobile phone data types: mobile phone data aggregated at the cell tower level; mobile phone data at the individual level, known as call detailed records (CDRs) data; and mobile phone data at the aggregate level, known as aggregated CDRs data.

The charts illustrate that mobile phone data aggregated at the cell tower level exceed mobile phone data at the individual and aggregated levels. A total of 58 studies (53% of the 107 primary studies) investigated mobile phone data aggregated at the cell tower level, while 35 studies (32% of the primary studies) examined mobile phone data at the individual and aggregated levels, usually called CDRs and aggregated CDRs, respectively. Almost 15% of the studies combined multiple data types; 7 (6% of the 107 primary studies) were survey papers in which the authors studied and reviewed all mobile phone data levels, while 10 (9% of the primary studies) utilized mobile phone data at the individual and cell tower levels. These studies used mobile phone data at these levels to investigate individuals’ social networks based on the calling information and to examine human mobility patterns based on the spatial and temporal characteristics. For the sake of simplicity and clarity, during the process of collecting information about the specific types of mobile phone data explored in the literature, we found that there was misunderstanding, confusion, and misuse of the correct terms for each mobile phone data type. For example, most studies refer to mobile phone data that are aggregated at the cell tower level as CDRs data, while CDRs data actually refer to mobile phone data at the individual level. For that reason, we devoted time to clarifying which terms were used for what types of data, finding that researchers and academics have referred to the vast majority of mobile phone data types as CDRs data. To solve this issue, we relied on three things. The first was the attributes that were utilized or investigated by a given study, where each mobile phone data type has different attributes. For example, mobile phone data aggregated at cell tower level have the following attributes: timestamp, user ID, and cell tower ID with the corresponding latitude and longitude coordinates. On the other hand, mobile phone data at the individual level (CDRs data) have the following attributes: caller and callee IDs; caller’s connected cell tower ID; callee’s connected cell tower ID; duration; and timestamp. Second, they showed whether the authors illustrated how the data were collected and generated, and third, what application was investigated by a given study; for example, mobile phone data aggregated at the cell tower level can capture users’ spatiotemporal change patterns based on the spatiotemporal information provided by this data type, which is thus mostly related to mobile phone data applications concerning mobility patterns. This procedure was tedious and time-consuming, but our efforts should help future researchers differentiate different types of mobile phone data and consider these points in the future.

### 3.5. Citation Count

Table 4 shows the top 13 most cited articles from the primary studies. However, the citation count is a time-variant variable, meaning that it will likely change over time. For the data collection process, the Science Citation Index, Social Science Citation Index, Web of Science, and Scopus are well-known tools for performing bibliometric analyses. However, the data used in this study were collected from Google Scholar, which provided higher citation counts than the aforementioned tools due to its ranking algorithm being more inclusive by including non-peer reviewed papers, working papers, and preprint papers. The total citation count of the 10 most cited papers in this domain is 3588.

Studies such as [1,3,6,20] have been the most influential due to, for example, Deville et al. [6] and Pei et al. [20] being the first to present the ideas described in their research. Pei et al. [20] solved the problem of inferring urban land use from mobile phone data by improving the existing classification of different urban land uses, while Deville et al. [6] was one of the first to use mobile phone data to map human population distributions instead of employing traditional datasets, such as censuses and surveys. A criminology study by [13] additionally employed mobile phone data to predict crime hotspots. Notably, Refs. [1,3] provided thorough overviews (research surveys) of how mobile phone data are used in different applications and domains. This table can be helpful for researchers and scholars as an index or reference for not only the most highly cited papers, but also for pinpointing papers on mobile phone data that they can use as starting points for further research in this domain.

### 3.6. Place of Publication

**Figure 8:**
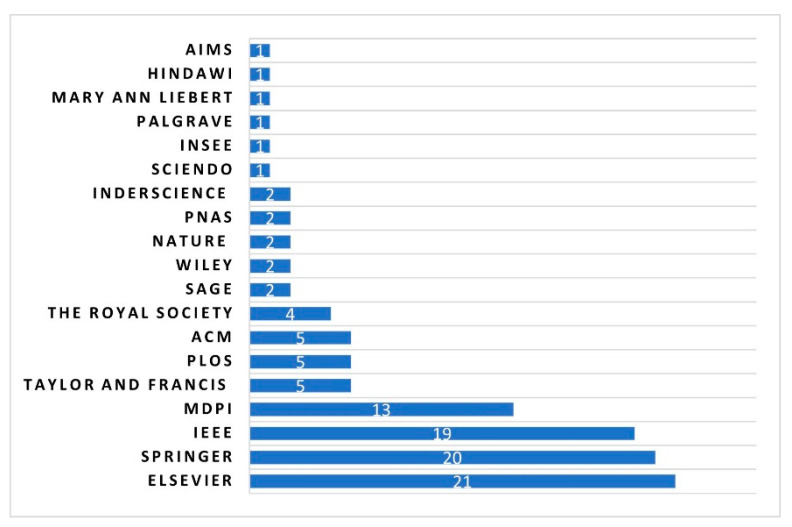
The distribution of publisher channels.

Figure 8 shows the number of selected studies grouped by place of publisher (journal publishing companies). It can be seen that the selected primary studies are chosen from a variety of different academic publishers. However, as the bar graph demonstrates, Elsevier, Springer, and IEEE have the highest share among 19 academic publishers with 56% (60 out of 107), which is not surprising. As a matter of fact, Scopus, which belongs to the same publisher as Elsevier, IEEE, and Springer, returned the highest share during the identification phase (the initial search result) with 75.2% (2855 papers out of 3796). We also observed that famous world-class publishers such as PNAS and the Royal Society, which are among the most prestigious and highly cited multidisciplinary research journals, are among the publishers in the mobile phone data domain.

### 3.7. Mobile Phone Data Methods and Problems

**Figure 9:**
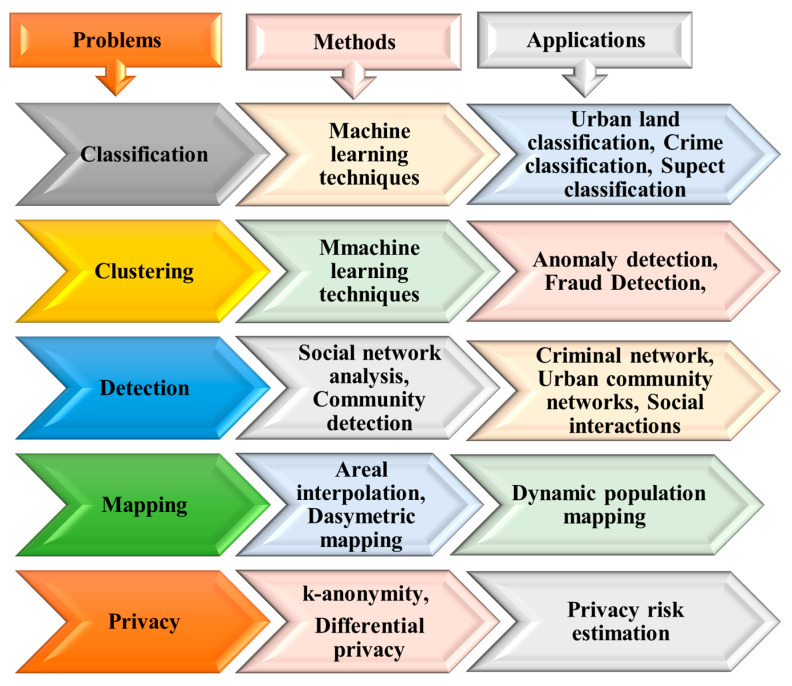
Comparison of different methods and problems used in mobile phone data studies.

Here, we detail mobile phone data problems and provide a comparison of different methods used to solve these problems (see Figure 9). Generally, mobile phone data problems can be divided into five main groups: classification, clustering, detection, estimation, and privacy problems. The relevant studies have mostly been related to clustering problems. Thus, many mobile phone data studies have been conducted to solve problems with clustering approaches in numerous applications. For example, in these references [20,37,38], inferring land use types was identified as a clustering problem. In References [39,40,41,42], the authors sought to identify users’ habits. In Reference [19], the authors clustered users based on their weekly patterns.

Social network analysis techniques and metrics have been widely used to solve problems related to the community detection problem (CDP), community structure, and social network visualization. For example, detecting criminal networks has been seen as a CDP. In References [28,29,43,44], the authors applied different community detection algorithms to detect criminal networks. Novovic et al. [45] applied community detection techniques to infer a correlation between human dynamics and land use. Moreover, Shi et al. [46] applied a community detection algorithm to detect the spatial interactions of urban social communities.

Classification problems have been examined in studies aiming to identify suspects. In References [9,47], classification algorithms were used to differentiate suspects from non-suspects. Another example of a classification problem is predicting crime hotspots. Bogomolov et al. [13] applied classification algorithms to classify crime hotspots into two classes, high or low crime levels. Moreover, Ref. [48] used algorithms to classify land uses.

Furthermore, k-anonymity techniques and approaches have been suggested as solutions to solve problems related to privacy risks and data protection, such as in [49,50]. For estimation problems and correlation analysis problems, statistical measurements, such as correlation coefficients and regression models, have been used; for example, Pearson’s correlation coefficients were used in [6,51], whereas Spearman’s correlation was used in [21,52]. Finally, areal weighting, dasymetric mapping, and Voronoi tessellation techniques have been used to solve problems related to spatial mapping and population mapping, such as in [53,54,55].

### 3.8. Analysis and Perspectives

**Figure 10:**
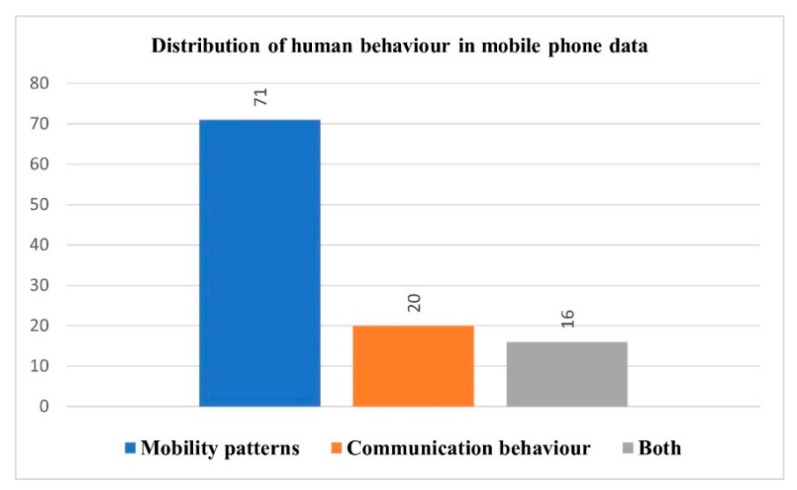
Distribution of analysis perspectives in mobile phone data.

The percentage of the selected articles studying human mobility patterns is higher than that of studies looking at communication behaviors. Figure 10 highlights this extraordinary growth in the number of studies that utilize mobile phone data to investigate human mobility and movement patterns, with these representing 66.4% (71 out of 107) of all studies, as compared to the mere 18.6% (20 out of 107) that used such data to study communication behaviors, and studies that investigate both human behaviors represented a further 15% (16 out of 107). This observation may be attributable to the fact that mobile phone data aggregated at the cell tower level were the most commonly investigated, as shown in Figure 6, which shows the distribution of mobile phone data types under investigation, and this type of data (mobile phone data at the cell tower level) reveals only information about human spatiotemporal patterns. As a result, several applications related to human mobility patterns can be derived from this data type, i.e., mobile phone data aggregated at cell tower level. This finding may also be explained by privacy concerns that restrict and increase the difficulty of accessing or acquiring mobile phone data at the individual level (CDRs data), which might contain sensitive details such as spatiotemporal trajectories and communication information about the receiving side of the communication, as opposed to mobile phone data at the cell tower level, which does not reveal communication details. Furthermore, due to difficulties seen in managing and processing CDRs data based on the nature of raw data, data cleansing and preprocessing, such as noise reduction and managing sparsity constraints, is required.

### 3.9. Network Visualization of the Co-Authorship Analysis by Country in Mobile Phone Data

**Figure 11:**
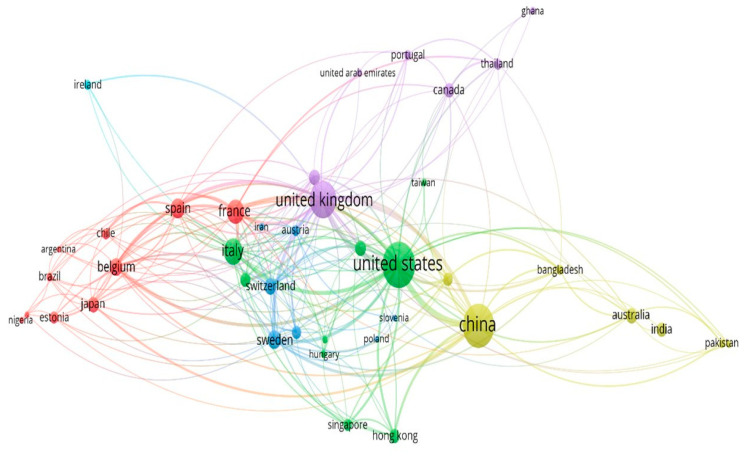
Network visualization of the co-authorship analysis by country.

This network in Figure 11 visualizes the worldwide co-authorship of the countries that have published articles on mobile phone data by evaluating the performance of the participating countries and the degree of cooperation between countries to produce papers on mobile phone data. Each node inside the cluster represents a country, and the node size refers to the publication weight, while the total link strength reflects the degree of co-authorship links to other countries. For example, the United States, China, and the United Kingdom have the largest proportion of publications in mobile phone data with 203, 181, and 131 publications, respectively, and the total number of co-authorship ties to other countries is 229, 136, and 187.

### 3.10. Co-Occurrence Network Visualization of Keywords in Mobile Phone Data Studies

**Figure 12:**
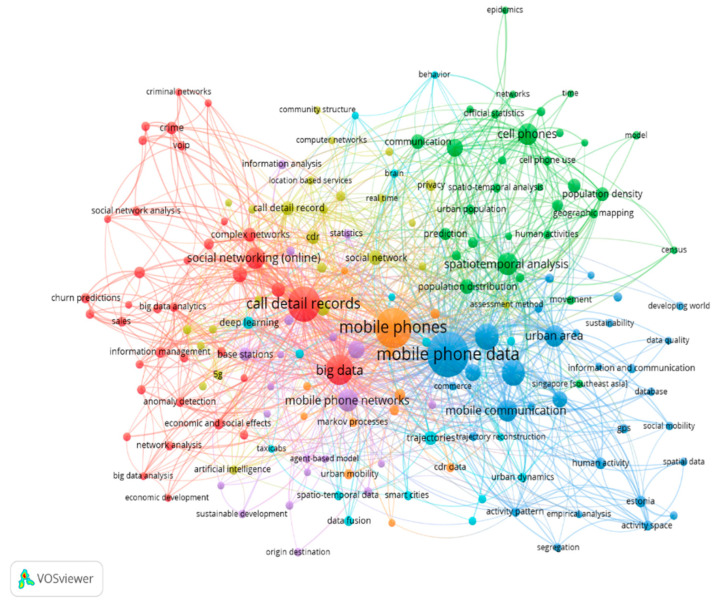
Network visualization of keywords in mobile phone data studies.

This section describes the construction of the keyword co-occurrence map, which is based on the co-occurrence data. The map in Figure 12 visualizes the co-occurrence network of the most frequently used keywords or search terms in mobile phone data studies. To further understand the relationships between different clusters within the network, each node has been made to represent its value or importance based on the occurrence weight of the node itself and the strength of its link with other nodes (each node represents a search term, links represent the occurrence of a pair of search terms, and the weight of the link is represented by the co-occurrence frequency of each pair of search terms). Node size refers to the frequency of the occurrence of a keyword (e.g., mobile phone data, detailed call records, etc.) in the selected publications, and it is measured by the number of articles that have used that keyword (or a corresponding term) in their list of keywords. The first cluster contains blue nodes and is the largest of all seven clusters. It depicts mobile phone data with a weight (occurrence) of 170 and 492 links, followed by human mobility with a weight (occurrence) of 84 and 219 links. The blue cluster includes the following human mobility pattern search terms: mobile phone data, human mobility, mobility patterns, mobile communication, urban area, and others. The second cluster contains red nodes representing terms related to mobile phone data types, such as CDRs data, and human communication behavior, such as social networking, social network analysis, criminal networks, big data, and economic and social effects. The third cluster contains green nodes that represent terms related to human mobility patterns and their applications, such as spatial–temporal analysis, population distribution and density, human activities, and geographic mapping.

## 4. Study Taxonomy

**Figure 13:**
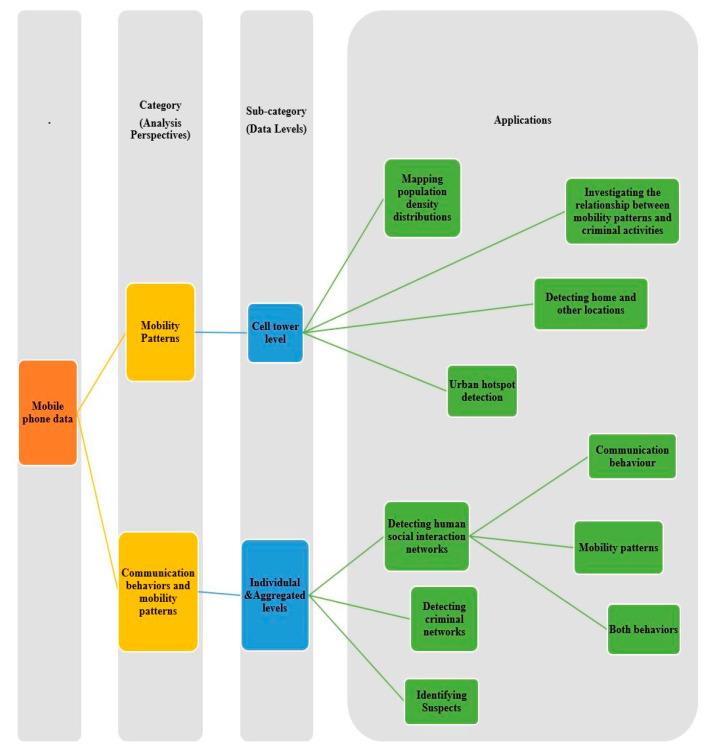
Taxonomy of research literature in mobile phone data.

This section describes the process by which the selected studies were organized and categorized into structured taxonomies in a way that helps address the research questions and sheds light on the current state of mobile phone data applications. A taxonomy is presented in Figure 13 that contains categories and subcategories of mobile phone data according to specific factors, including the analysis and processing techniques (analysis perspectives), the dataset level (i.e., individual, aggregated, cell tower), and types of applications. Generally, mobile phone data are utilized in the analysis of human communication behaviors and mobility patterns; thus, the taxonomy is categorized according to aspects related to the analysis perspectives of mobile phone data. Since the analysis of mobile phone data occurs at three different levels, which are the individual, aggregated, and cell tower levels, a new classification is made according to these levels. Similarly, the processing techniques used to analyze mobile phone data on each level embrace numerous applications that also require subcategorization as a new classification.

### 4.1. Mobility Patterns (Main Category): The First Leg

The first leg of the study comprises mobility patterns, which are further classified into one of the mobile phone data types (levels), in this case, mobile phone data aggregated at the cell tower level. This leg discusses the studies that use mobile phone data aggregated at the cell tower level. Such data store records that detail the user ID (caller ID), the timestamp (e.g., call date, call time), and the location data (cell tower ID), where each record is geolocated based on the nearest BTS. These details allow us to capture users’ spatiotemporal change patterns extracted from spatiotemporal information collected from this type of data. Thus, mobile phone data aggregated at the cell tower level have been used for several applications related to human mobility patterns, such as estimating populations, identifying home and work locations, and identifying land use types.

Mobile phone data at this level are usually used to capture human mobility patterns since only spatiotemporal information is recorded. Thus, the details at this level allow for the estimation of a population in a certain block or area based on the phones that are connected to the cell tower. Identification of visitors to and residents of an area is carried out by identifying nearby cell towers that the mobile devices are connected to most of the time. Furthermore, the activities of mobile phone users within a given geographical location can be captured by the cell towers connected to them, and changes in the location of mobile phone users from one place to another can allow them to be identified as residents or visitors based on human activity, represented by spatiotemporal characteristics obtained from mobile phone data. These spatiotemporal characteristics that explain human activities provide a wide array of applications, which will be discussed here.

Mobile phone data at the cell tower level cannot be used to investigate and study human communication behavior and social communication patterns because they do not contain information regarding calling patterns that illustrate details of the other side of the communication. A full description is added later for the second leg when discussing applications used for mobile phone data at the individual and aggregated levels, usually called CDRs and aggregated CDRs, respectively. Thus, this leg mostly discusses human mobility patterns based on mobile phone data aggregated at the cell tower level.

#### 4.1.1. First Application: Estimating and Mapping Population Distributions

Many aspects of human activities are related to human mobility patterns, and investigating these patterns has become a common use of mobile phone data; this can be seen clearly in the number of applications derived from this analysis perspective based on the spatiotemporal characteristics that can be extracted from mobile phone data that cover multiple aspects of human life activities. Importantly, this includes estimating and mapping population distributions. Mobile phone data have been used to estimate population densities by mapping the hourly dynamics of population based on spatial-temporal trajectories extracted from mobile phone data. To map the population’s presence at a cell tower, Deville et al. [6] applied an interpolation method of spatial mapping known as areal weighted interpolation (AWI), which allows the interpolation of coverage areas’ spatial division and its attributes through areal intersection with spatial units, such as blocks or administrative areas. In this manner, the areal weight of a census block can be intersected with the cell tower coverage of a mobile network. This study led to further discoveries based on such applications. Sakarovitch et al. [56] aimed to estimate resident populations by using Voronoï tessellation, which partitions the geographical space of cell tower coverage into Voronoï polygons. By applying dasymetric mapping methods to enhance population mapping on a more fine-grained spatial scale, Ref. [53] applied a two-step floating catchment area method (2SFCAe) and land use regression (LUR).

However, as these methods only consider mapping populations based on spatial distribution, to improve the mapping of the spatial distribution of cell towers with respect to population and thus to map population dynamics, a more fine-grained spatial and temporal scale is required; various researchers [55,57,58] have thus applied dasymetric interpolation methods to map population distribution by integrating this with a temporal perspective. The aim of such work is to use multi-temporal function-based dasymetric (MFD) interpolation to enhance the accuracy of the spatiotemporal resolution of population dynamic distributions by capturing temporal patterns. However, Liu et al. [59] criticize previous work in mapping dynamic populations due to their failure to estimate a population distribution at a fine temporal scale due to capturing the temporal patterns of the population only over a given time period. Thus, they aimed to map population dynamics at hourly intervals by reconstructing time series trajectories of hourly population density. In their quest to enhance the accuracy of mapping population density distribution effectively, Ref. [60] determined that a lack of ground truth data for the dynamic population density distribution over various time scales might affect the estimation of a population at a finely grained temporal resolution; they thus used the Tencent positioning dataset with fine-grain temporal resolution as ground truth data for training in a deep learning model using a deep convolutional generative adversarial network (DCGAN).

However, Salat et al. [54] criticized previous methods because they required a large number of finely grained data sets in order to train a given model, such as census and satellite data. This is especially the case in some developing countries where census data are not always available to validate the models; thus, the authors sought to provide a model without requiring training datasets by applying a hierarchical clustering method (hierarchical cluster analysis). References [61,62] sought to solve these problems related to estimating the population density, such as data heterogeneity and multiple sources of mobile network operator (MNO) data. They performed this by proposing two novel methodological frameworks which they designed to correlate multiple mobile phone data sources (location area-level data, CDRs, aggregated CDRs, and mobile phone data) from multiple MNOs based on data fusion models and joint analysis techniques.

Taking this idea further, based on a similar aim to map and estimate population distribution, Shi et al. [63] not only attempted to estimate population density distribution but also strove to investigate the correlations between population density distribution and public service facilities such as retail stores, businesses, hotels, culture and art facilities, and parks. The findings of their study showed that the distribution of public service facilities is strongly linked to population density during the day (daytime population).

#### 4.1.2. Second Application: Investigating the Relationship between Human Mobility Patterns and Criminal Activity Patterns

This application covers studies examining human behavioral activities as reflected in spatiotemporal mobility patterns extracted from mobile phone data and their associations with crime patterns. Empirically speaking, estimating how people move, measuring their presence at a given place, measuring population risks, and estimating the flow of the general population to provide information about criminals’ movements—all these measures have been reconstructed or derived from spatial-temporal characteristics in mobile phone data as part of investigations into their relationship with crime patterns, as well as to develop a better understanding of spatiotemporal patterns of crime. References [13,14,64] were some of the first studies that investigated the correlation between human mobility patterns and criminal activity patterns in the mobile phone data domain. Traunmueller et al. [64] aimed to observe such a correlation based on testing Jacob’s hypothesis, which suggests that high population density and population diversity (age diversity, ratio of visitors, and ratio of residents) reduce violent crime rates, and the results show that the relationship between crime activities and the diversity of age and ratio of visitors was negatively correlated. Bogomolov et al. [13,14] performed one of the first studies to investigate correlations between human behavioral activities as depicted in spatiotemporal patterns from mobile phone data and criminal activities. The authors used this data as a proxy to measure people’s presence at a given place to predict the relevant crime levels (classifying crime levels) in terms of “low crime levels” or “high crime levels”. These studies opened up the type of data used in such applications, and [21,51,65] then went a step further by using mobile phone data as a measure to estimate the ambient population (population at-risk). In the [21] study, the authors measured the ambient population to investigate its relationship with crime rates, with results that showed a strong correlation between the ambient population and theft crimes, based on identifying “people who might commit theft”. Similarly, Ref. [51] estimated the ambient population as an alternative measurement of the population at risk to investigate the effects of the ambient population on the spatiotemporal patterns for migrants and natives in terms of violence committed by migrants and natives, and the results show that the ambient population has a positive relationship with migrant violent crimes. Finally, Ref. [65] aimed to examine the relationship between the ambient population and the spatial crime pattern of larceny-theft, with results showing that the ambient population has a positive link with larceny-theft crimes.

Instead of estimating the ambient population as in previous studies, References [52,66] investigated the correlations between exposed population-at-risk (population at risk of exposure to violence), which may be a mix of criminals and victims, and temporal–spatial patterns of violent crime in public to determine their impact on violent crime in public spaces. Haleem et al. [52] aimed to evaluate the influence of exposed and ambient populations-at-risk on violent crimes associated with the nighttime economy (NTE). Lee et al. [66] built on the same notion of “exposed population-at-risk”, with the addition of the spatial and temporal characteristics of violent crime in public spaces. Finally, Song et al. [67] attempted to determine whether the daily mobility flows of the general population could provide a template for the daily mobility of criminals.

#### 4.1.3. Third Application: The Detection of Homes and Other Meaningful Locations

Many studies have focused on identifying home and work locations, which can be part of the analysis of mobile phone data. Not all studies were primarily focused on the detection of home and work locations, but during the analysis phase, the detection of home and work locations may have been required for preprocessing prior to further analysis. This process has been widely followed with mobile phone data in many studies [5,12,13,18], while other studies have mainly focused on detecting home or work locations [35,68,69].

Human mobility patterns extracted from mobile phone data have been used to model daily human activities (for example, home, work, shopping, etc.) by parsing trajectory features from spatiotemporal information into fixed locations. This indicates where people conduct their activities or the locations where the most activity takes place. Therefore, daily activity patterns based on mobile phone data can identify and estimate home and work locations or other meaningful locations. Kung et al. [35] aimed to detect home and work locations based on human mobility patterns. Two criteria were chosen to define home and work locations, namely, the home location was identified as the most frequently visited location during nighttime, and the work location was the most frequently visited location during daytime hours. Empirically, identifying a person’s home means that a single cell tower is allocated as their home location, so the most frequently used cell tower location during the night hours, for example, 7 p.m.–7 a.m., is the approximate location of residence. Tongsinoot and Muangsin [68] identified the home detection by correlating mobile phone data with Internet data usage containing attributes such as mobile numbers, timestamps, upload volume, download volume, cell tower ID, and network ID. This was conducted to improve the detection of home and work locations, claiming that previous identification methods are based on the time or duration criteria, where the proportion of staying time is calculated to estimate the location. Vanhoof et al. [69] aimed to improve home detection by defining five home criteria based on calling activities and mobility patterns.

#### 4.1.4. Fourth Application: Urban Hotspot Detection

This part highlights scholarship that uses mobile phone data to detect urban hotspots (hotspots refer to regions with higher concentrations of people, the most congested places, or high-intensity crime areas) based on human mobility patterns. Louail et al. [34] sought to detect urban crowd hotspot areas (areas considered to be dense) in 31 Spanish cities. They performed this by extracting spatial–temporal characteristics, such as aggregating every hour (because hotspots fluctuate over time as a result of human mobility patterns) the total number of mobile users in each cell tower, which helps in turn to estimate population density. After depicting user density based on human mobility, the authors then established a threshold to identify hotspots by using a non-parametric method based on the logarithmic derivative of the Lorenz curve. The threshold density population describes each cell i (cell size is between 500 m and 2 km) with a density of users larger than the threshold δ for the density ρ(i, t) > δ as a hotspot cell at time t, while Yang et al. [70] set out to detect two types of urban human dynamics hotspots—convergent and dispersive hotspots—in the city of Shenzhen, China. In order to identify human mobility hotspots, they applied an unsupervised clustering algorithm, the X-means algorithm, statistical analysis, and kernel density estimation (KDE). Similarly, the KDE method was used by Ghahramani et al. [71], who aimed to detect hotspots in Macau, China. However, the authors extracted characteristics of calling behaviors to illustrate urban population density, such as the frequency of calls at different timestamps and the duration of calls, along with spatial and temporal characteristics such as spatial objects referring to cell towers.

### 4.2. Communication Behaviors and Mobility Patterns (Main Category): The Second Leg

The second leg comprises communication behaviors and mobility patterns at the individual and aggregate levels. This section thus discusses what applications remain to be derived from mobile phone data at the individual and aggregate levels, and what human behavioral patterns may be captured by these data types.

Mobile phone data at both the individual level and aggregate level can be used to investigate and study human communication behavior and social communication alongside mobility patterns due to the fact that mobile phone data at both the individual level and aggregate level contains both communication information and spatial–temporal information. Individual data do contain details that reflect attributes such as caller ID, callee ID, caller’s connected cell tower ID, callee’s connected cell tower ID, duration of call, and timestamp, which allows the development of applications related to communication behaviors such as the mobile social networks (detecting social networking), the detection of criminal relationships, inference of social ties, and various applications related to human mobility patterns, such as the identification of suspects based on spatiotemporal characteristics, and the detection of criminals based on their calling patterns and mobility behaviors, which all will be discussed in this section. A full description of the applications derived from mobile phone data at individual and aggregated levels is thus offered in the next section.

#### 4.2.1. Social Network Applications

Mobile phone data’s application to the investigation of mobile social networks is a solid and self-sufficient topic. The study of human sociality using mobile phone data has evolved into a distinct field of study that gives insight into the dynamics of human social networks [30], which explains the rapid expansion in the volume of such studies. Mobile phone data have thus been used to study a huge range of human sociality-related topics across various applications, including the investigation of social ties, the inference of relationships, the detection of social networking communities, and the detection of temporal or spatial social networks based on spatiotemporal characteristics.

#### 4.2.2. First Application: Detecting Human Social Interaction Networks Based on Spatiotemporal Mobility Patterns

This application focuses on studies that use CDRs to identify social communities based on spatiotemporal mobility patterns, or, in other words, using mobility patterns as a means to detect communities. Shi et al. [46] constructed human social network interactions in a manner that aimed to discover spatiotemporal interaction communities arising from spatial human mobility patterns extracted from spatiotemporal information in CDRs data, such as the identification of the most frequented locations of users, identified as homes and workplaces, based on each user’s most active cell tower. To achieve this aim, the authors applied two methods: the Newman method and the Moore community detection algorithm, which detects social communities and uses the kernel density estimation method to visualize the spatial distributions of different communities. Truică et al. [72] aimed to detect or cluster groups of nodes that reflected social interactions based on spatiotemporal information (mobility patterns) by applying the Louvain algorithm, a well-known community detection algorithm, while Xu et al. [73] aimed to detect communities across faculty members and students in a virtual campus mobile network based on spatiotemporal patterns of users’ trajectories. Lind et al. [74] built social networks that aimed to detect spatial–temporal interaction communities based on human mobility patterns extracted from CDRs data; however, the authors also extracted one additional attribute from CDRs data, Internet usage, based on the fact that detecting communities based on just SMSs and phone calls limits the registration of spatiotemporal information to cell tower contacts; adding internet usage to represent interactions thus increases observed user events by allowing the visualization of additional areas (spatial information) based on user-triggered events, such as browsing or accessing the Internet. Sumathi et al. [75] aimed to build a social events network to detect and estimate the number of participants attending the Indian Institute of Science in Bengaluru based on their mobility patterns.

#### 4.2.3. Second Application: Detecting Human Social Interaction Networks based on Human Communication Behaviors

This section discusses studies where human communication behavior is used as a means to construct social networks. Schläpfer et al. [36] constructed social networks of human interactions based on communication behavior extracted from CDRs data, such as the total number of contacts, call volume, and number of calls, where subscribers (contacts) are the nodes and the call volume and number of calls are measures of reciprocity to quantify social ties between nodes. Their aim was to investigate the relationship between the size of the city and human social interactions, which in turn scale superlinearly with the city population size. Filipowska et al. [76] aimed to build user social profiles based on call information, including the number and duration of phone calls, to visualize social network activities among groups of users to help differentiate or classify relationship levels based on defining weak or strong ties between individuals. Reference [77] constructed students’ social networks based on communication behaviors represented by their calling characteristics, such as the total number of calls and SMSs as well as call duration. Their aim was to construct students’ social networks based on identifying chronotypes, such as owls (evening-active) and larks (early risers and early sleepers). In this process, degrees were assigned to each node and weights were assigned to each link in order to assess network structures. Yu et al. [78] also constructed social networks of friend relationships based on communication behaviors extracted from calling information that included the total number of calls and SMSs, along with call duration, timestamps, and other measures, by applying a semi-supervised algorithm. Their aim was to classify user relationships based on the strength of social ties between two classes, “friends” and “non-friend”. Similarly, Gaito et al. [79] built social networks to visualize human social interactions based on communication information that included the number of calls and SMSs, call durations, and call frequency. Their aim included investigating which communication channels, as represented by phone calls and text messages, users preferred for their interactions.

#### 4.2.4. Third Application: Inferring Social Network Based on Mobility Patterns and Social Interactions

Other studies combined both types of human behaviors, such as [5,80], both of which sought to capture macroscale patterns of mobility and social interactions. They studied the interplay between human mobility patterns and human social interactions to investigate how human mobility patterns influence social interactions.

Deville et al. [80] aimed to capture any relationships between human mobility and social networks, based on combining three different mobile phone datasets simultaneously to capture two perspectives on human behavior, defined by human mobility and social networks. The results revealed that these two behaviors are not independent, as there is a strong relationship between human mobility and communication patterns within social networks: as distance increases, the average number of fluxes in social interactions increases (number of calls) given the same volume of mobility fluxes (number of jumps between two locations). Phithakkitnukoon and Smoreda [5] investigated the interplay between human mobility and sociality (in terms of social tie strength) by extracting human social behaviors from calling information such as the daily number of calls made and received, call duration, and human mobility patterns as extracted from spatiotemporal information such as the number of locations a person visited in a time period, the travel distance range, and the degree of variation. Finally, Morales et al. [81] constructed an ethnic interactions network based on mobility and communication patterns by correlating two datasets, in which the first contains calling information and the latter contains spatial–temporal information. The network aims to detect different ethnic and religious groups in Ivory Coast by mapping each community to its geographically closest ethnic group. To achieve this goal, the authors applied community detection techniques such as the Louvain community detection algorithm and a K-means clustering algorithm.

#### 4.2.5. Fourth Application: Suspect Identification

This application arises from studies related to identifying suspects (people who are thought to be involved in certain criminal activities), based on detecting suspicious activities and movement patterns of all parties involved by examining the digital traces left by mobile phone devices that depict communication behaviors and mobility patterns. Digital traces left by people at locations where a crime has taken place, for example, can reveal a representative sample of the population present at a crime scene at a given time. Table 5 shows different features and analytical perspectives used to identify suspects.

#### 4.2.6. Fifth Application: Detecting Criminal Networks

This application is drawn from studies that utilize CDR data to detect criminal networks based on communication behaviors and mobility patterns.

With regard to the detection of criminal networks based on communication behaviors, Ferrara et al. [10] proposed a forensic analysis system named LogAnalysis, whose conceptual framework aimed to detect the most influential criminals in a criminal organization by applying social network analysis (SNA) tools and metrics such as degree centrality, closeness centrality, and “betweenness” centrality to identify both influential members and less-involved members of criminal networks and to quantify the degree of the relationships between vertices. Similarly, Refs. [11,28,29,43,44] proposed multiple forensic systems to detect criminal networks based on the calling characteristics of criminal communication behaviors, including outgoing and incoming calls between two identified vertices (criminals) and the maximum and minimum numbers of incoming or outgoing calls and messages. The SIIMCO system created by [11] aimed to detect lower-level criminals and their immediate leaders in a criminal network, as these are the most likely to be arrested, while the IICCC by [43] and CLDRI by [44] systems aimed to detect high-level criminals, identified as the most influential members in a criminal organization. ECLfinder [28] similarly aimed to detect and classify both high-level and lower-level criminals in a criminal network. Agreste et al. [29] aimed to uncover the underlying structure of Italian Mafia gangs and detect their key leaders.

Other work focused on the detection of criminals based on mobility patterns: Griffiths et al. [12] aimed to detect mobility patterns within specific terror networks (UK-based Islamist terrorists) by extracting various spatial and temporal features such as the locations most frequently visited by each criminal as reflected in their phones’ connections with each cell tower, which would also allow the measurement of the relevant distances between criminals’ home locations, crime locations, and other time-stamped locations.

## 5. Research Questions

The review introduces two research questions to cover the absence of data in the literature and what existing literature lacks in the fields of criminology and urban sensing.

### 5.1. RQ1: What Are the Current State-of-the-Art Methods and Techniques Regarding the Use of Mobile Phone Data to Identify Suspects and Predict Crimes?

Before reviewing the state-of-the-art methods and techniques that employ mobile phone data for the identification of suspects and prediction of crimes, we first give a brief discussion on why such data can be seen as a sensor for human activities and mobility in the context of criminology.

Mobile phone data contain different kinds of digital traces, such as mobility traces and communication traces, which can be used as evidence in criminal investigations [89]. Therefore, the digital traces left by a large number of mobile devices provide valuable information that facilitates the understanding of human behavior and mobility in the context of criminology, such as the prediction and identification of crimes and suspects. For example, Griffiths et al. [12] analyzed the mobility behaviors of criminals based on the digital traces they left at home and other meaningful locations, such as the crime scene. The mobility traces of criminals were identified by cell tower locations where they previously received a call. The traces were then analyzed to determine the regularities in the criminals’ movements and to investigate whether the movements were not random. The authors subsequently concluded that there is a high degree of spatial regularity in the criminals’ movements.

We report the state-of-the-art methods and techniques concerning the use of mobile phone data in identifying suspects and detecting criminals. A particular focus is given to existing scientific literature that has explored the use of mobile phone data in the context of criminal behavior from people- and place-centric perspectives. Our taxonomy of crime study concerning mobile phone data identified three applications: suspect identification, criminal network detection, and investigating the correlation between human mobility patterns and spatiotemporal crime patterns.

#### 5.1.1. The First Group of Applications Deals with Using Mobile Phone Data to Identify Suspects

A suspect is defined as an individual who is suspected to be involved in a crime [47] based on the digital traces left at the crime scene. In criminal investigations, location-based mobile phone data can be used to indicate the presence of suspects in an area at a certain time when a crime has taken place, whereas communication traces can be used to identify accomplices in a criminal activity. As examples, References [82,84] collected mobility traces left at crime scenes to determine suspects’ positions and presence at the crime scenes.

At the identification phase, the literature has shown that researchers used several parameters and attributes to determine suspects (e.g., outgoing calls, incoming calls, the start time of the call, location, duration, the number of calls made, and messages received). For example, in [9,83] methods, the researchers extracted communication information such as “outgoing calls”, “incoming calls”, frequent callers”, and “maximum duration” to identify suspects. However, Reference [87] extracted spatiotemporal information along with communication details to identify suspects.

#### 5.1.2. Suspect Identification Models Can Be Divided into Unsupervised and Supervised Models

Unsupervised models use unlabeled data and subjective definitions for the identification of suspects (e.g., “suspects are those who contacted previously contacted criminals and also made calls nearby the crime scene”).

Supervised models use historical data where each user is labeled as suspect or non-suspect and try to find patterns in phone call data records that distinguish between those who were historically selected as suspects and non-suspects.

Khan et al. [85] used CDR data of various suspects and victims in order to extract associations between pairs of telephone numbers that can point out a few correct directions for identifying the most likely correspondence between suspects and victims. The methodology was based on the idea that frequent calls and the duration of calls may be indicative of a criminal–victim relationship.

The technical implementation was conducted using a combination of Hadoop (a framework for distributed processing of large data sets across clusters of computers) and Hive (a data warehouse architecture for querying data stored using Hadoop). The choice of tools was justified by the widely known efficiency of these tools for mining big data and by the security of Hadoop, which is important due to the use of highly confidential data. Even though it is a “simple implementation”, some important weaknesses are worth mentioning. There is no evidence that frequency of calling or maximum call duration are helpful in identifying actual criminals, as authors do not validate their model against ground truth data. They used only a very limited set of call features, not including spatiotemporal characteristics, to identify suspects. The use of location data would have been useful in placing the suspects and their accomplices at the crime scene.

In Reference [83], the authors used CDRs data to identify links that exist among criminals and anti-social elements. Their analytic approach was based on the idea that anti-social elements have their own network of contacts, and the identification of those closely linked to previously convicted people is helpful in shortlisting suspects. That is why their network analysis methodology was based on graph theory as a tool for identifying otherwise hidden relationships.

While the authors demonstrated an actionable graph-based decision support tool for streamlining inference that would otherwise be difficult to achieve using slicing and dicing data in spreadsheets, the study has some weaknesses. The approach relies on looking for exact matches in long-term historical data and thus makes the unrealistic assumption that suspects do not change their mobile devices after communicating with convicted criminals, which looks like very incautious behavior for experienced criminals.

Authors in Reference [9] proposed a supervised machine learning method to identify suspects. The task was to classify users into suspects and non-suspects. The researchers turned the CDR-level dataset (13 million rows) into a user-level dataset (10,000 rows) through data aggregation and feature generation. As a result, each user was characterized by 30 discrete features derived by discretizing such features as the number of calls made, the average duration of calls, the proportion of calls and text messages, etc. A targeted Bayesian network learning (TBNL) model was applied that resulted in a descriptive network in which the selected features and their interactions were used to discriminate between positive (e.g., “suspects”) and negative (e.g., “non-suspects”).

The model was validated using 10-fold cross-validation, where the sample of 10,000 users was split into 10 random folds of 1000 users each, and every time, 9 folds were used for training and 1 fold was used as a holdout sample for testing purposes.

The main strengths of the study are the demonstration of the proposed model’s strength relative to several competing algorithms and the fact that the model results in actionable empirical facts about factors that increase the probability of being a suspect. The main weaknesses of the study are as follows: The authors demonstrated that the text-to-call ratio is substantially lower for suspects than for non-suspects in the late morning hours but did not provide the same type of interpretation regarding other predictors. The cross-validation procedure was used for optimizing model parameters, feature selection, and measuring its performance, which could inflate performance metrics. This could have been avoided by keeping around 10% of the dataset for final model testing.

Hassan et al. [47] applied a Graph Convolutional Network model (GCN) in order to identify suspects from non-suspects. The authors built a straightforward undirected graph (G), represented by the input matrix *A*, which was to be applied to two-dimensional convolutional layers. Graph *G* contains six nodes, with the features of each node used to classify criminals from non-criminals. Hence, the authors performed a semi-supervised classification method on a small number of labeled data to train the classifier (seed nodes belong to convicted criminals to help train the classifier). The output then is a single binary for each node, indicating whether the corresponding node is predicted to be a suspect or not. Although the proposed method has yielded promising results, it is not without limitations. To begin with, CDRs were fully employed; therefore, the communication information only featured the node. Second, because the resulting network of CDRs data is rather sparse, modeling the network in the context of seed nodes may require domain-expert knowledge. Methods in deep learning are usually effective as long as they require large numbers of data. Thus, Hassan’s model produced a sparse network due to the limited training sample.

#### 5.1.3. The Second Application Deals with the Detection of Criminal Relationships Based on Communication Behaviors and Mobility Patterns

Once the suspects in a crime have been identified, it is important to investigate the roles that each criminal plays within a specific network. Connecting a suspect to other perpetrators and understanding their relationships with criminal networks is difficult, and thus the use of CDRs data has been increasingly exploited by social network analysis (SNA) tools and metrics, including degree centrality and betweenness centrality, all of which can be used to identify influential members and low-level members of criminal networks.

#### 5.1.4. The Construction of a Social Network from Mobile Phone Data

A graph (or network) can be used to model mobile phone data (Graph 𝐺 = (𝑉, 𝐸). A graph contains various nodes (or vertices) that represent different mobile phone users, and the edges 𝐸 represent text messages and calls between two individual users.

Studies on social networking cover many topics, including community detection, social network structure, and measuring network modularity. Partitions or clusters of nodes in a graph are typically known as communities in network investigations. Communities can be structured in two ways: non-overlapping structuring, in which nodes belong to only one community, and overlapping structuring, in which nodes can be part of multiple communities. Several researchers have used social network analysis tools to solve the community detection problem [45,46,90]. SNA tools can also help investigators understand the hierarchical structure of criminal networks since it is difficult for forensic analysts to determine who belongs to a criminal organization and the relationships that exist within it. Thus, SNA can be harnessed to determine the relations and interactions between criminals by reconstructing the communication relationships that are obtained from mobile phone data as a network, where a node represents a criminal and an edge represents a communication (i.e., a phone call or a message). Using this approach in the analysis of criminal networks allows the investigators to understand the hierarchy and structural properties of the network.

#### 5.1.5. Detecting Criminal Networks Based on Communication Information

In this section, research that has used social network analysis tools and measures to detect criminal networks based on mobile phone data will be presented. 

In the relevant works of the literature, a collection of SNA techniques and algorithms have been used to detect and probe criminal networks. These approaches and algorithms have primarily been used to solve problems relating to community detection, while statistical metrics have been used to analyze relationships between vertices and assess structural centrality in networks. Several researchers [10,28,43] have developed many detection methods based on communication information extracted from mobile phone data to detect communities in criminal networks. Moreover, these researchers have employed the same analytical method used to investigate criminal networks (namely, social network analysis) but with different detection algorithms.

For instance, Ferrara et al. [10] proposed LogAnalysis, a criminal investigation expert system, to detect criminal networks. This system incorporates a well-known detection algorithm called the Girvan and Newman (GN) algorithm. They opted to use the GN algorithm due to its capacity to identify edges in networks lying between communities (when edges are less central, they are most likely to fall ‘‘between” communities). Subsequently, the system removes these edges, leaving the communities behind. The researchers have used this system to identify interconnected nodes that belong to different clusters and gradually remove them, which disconnects the clusters and ultimately reveals the community structure. Then, edge-betweenness and centrality metrics were calculated. The measurements focus on the less central edges, where the edges are most ‘‘between’’ communities. This is more effective than using a measure that focuses on the central edges. In the experimental setup, 381 nodes and 428 edges were identified. After the mobile phone network was configured and the detection algorithm was applied, a total of 16 communities were identified. The key objective was to identify edges from interconnected nodes that belong to different clusters (different communities) and progressively remove them. Therefore, the edge-betweenness centrality measure was incorporated in the algorithm, which facilitated the removal of 28 edges and the development of a community consisting of multiple groups (after each node is assigned to one cluster). The findings also reveal that all vertices are linked to a central vertex, which serves as a hub and generates a centralized network. This happens because GN is greedy in its approach to clustering and focuses on collecting vertices in the network [11]. To perform this, a number of rules are followed, after which vertices are merged to create a coherent division of the criminal community structure. Therefore, if the central vertex is removed, a hierarchical network can be created, which, in turn, enables subgroups to be identified through their interactions with other group members.

After the researchers had identified the social criminal network, they plotted it on a graph using a visualization tool. Visualization plays a major role in increasing investigators’ comprehension of the complexity of the network; thus, it is a useful tool for visualizing and presenting complicated networks. They tried three different visual layouts, namely, the node–link diagram, the convex hull, and the force-directed layout.

Following that, Agreste et al. [29] worked with Italian law enforcement to collect mobile phone data that revealed the communication details of the Sicilian Mafia group. This work was similar to that of [5] in terms of detecting and structuring criminal networks but different when it came to describing how the criminal network functioned. The researchers created two networks, one of which was based on the mobile phone data and thus contained the identities of 1716 suspects (vertices) and 8481 contact logs in the form of phone calls, SMS, MMS, etc. (edges). On the other hand, the second network was based on the relationships between various individuals involved in criminal acts. The two networks were merged through an aggregated network that enabled all pairs of nodes to be connected by an edge in at least one network. Meanwhile, the results show that there are several criminals who can be identified by correlating mobile phone data with crime data.

Other criminal detection systems based on social network analysis are carried out by adopting Prim’s Minimum Spanning Tree (MST) algorithm in [28], the Concept Space Approach (space algorithm) in [11], and Blondel’s algorithm in [47]. The most significant variation between these systems are the metrics and measures used to identify key members of criminal networks (see Table 6 for more details).

#### 5.1.6. Detecting Criminals Based on Spatiotemporal Information

On the other hand, studies have investigated the use of spatiotemporal information to identify criminal relationships and activities.

For instance, Hassan et al. [47] identified suspects by monitoring the spatial–temporal movements of criminals, while the authors in Reference [12] carried out cumulative frequency analysis using various statistical functions, including cumulative distributions and cumulative probability distributions, to determine whether criminals have routine activity spaces. Thus, the authors extracted spatiotemporal characteristics of criminals, such as the distances between their homes and safe houses (i.e., bomb manufacturers or armories) and the most commonly visited locations. The findings indicated that the criminals frequented particular areas, with most of their activity clustered between their home and safe house (crime location). Ultimately, this implies that criminals do not select targets randomly and that their movements are routine and steady.

Furthermore, Feng et al. [51] studied spatial variations in crimes perpetrated by both native and migrant criminals by correlating multiple mobility datasets, including offender data, mobile phone data, and points-of-interest (POI) data. The authors selected anchor points to identify criminal spatial patterns as well as to understand what motivates criminals to carry out crimes in the proximity of their homes. The findings revealed that offender anchor points are more prominent in native violent crimes than those perpetrated by migrants. This is because criminals’ homes and crime scenes share similar spatial patterns. This means that native offenders are much more likely to use their homes as anchor points when selecting targets for their crimes. On the other hand, migrant criminals are more likely to be impacted by crime attractors, crime generators (such as bars, clubs, etc.), and areas with vast populations.

The studies of both [12,51] explored criminal anchor points. Anchor points may be residences, workplaces, or any significant area that a criminal leaves to carry out a crime. Anchor points play a critical role in identifying places of importance to criminals and in detecting the spatial mobility of criminals. This is because criminals typically target areas near their residences to commit crimes. In other words, the probability of committing a crime decreases as one moves further away from their anchor points; thus, violent crimes tend to take place near the offender’s anchor points.

To summarize, some studies detected criminal networks by analyzing communication behaviors based on extracting call information, whereas other studies analyzed criminals’ activities based on spatial–temporal mobility patterns, as presented in Table 6; however, detecting criminal activities by taking into account both criminal communication behavior and mobility patterns may be extremely useful [4].

#### 5.1.7. The Third Application Deals with Using Mobile Phone Data to Investigate Human Mobility Patterns and Spatial–Temporal Crime Patterns

The spatiotemporal patterns of crimes can be determined by extracting human routine activities and mobility patterns from mobile phone data [52] and then examining the correlations between the human dynamics and crime data [64]. Accordingly, mobile phone data have been widely used in crime analysis and predictions to identify crime hotspots [14], investigate the relationship between ambient population and crime hotspots [21], and measure population density at a certain place based on the number of mobile devices connected to a given cell tower located in the area where a crime has taken place.

Unlike previous applications, here, a large sample of the population is considered as a measure to investigate the relationship between human dynamics and crime patterns. Such a measure helps gain further insight into exploring whether mobility patterns of the population can help predict where criminals commit crimes [67] or serve as a measure of ambient population-at-risk [66]. Estimating the correlation between population dynamics and crime patterns was earlier investigated by Bogomolov et al. [13], who extracted users’ locations to estimate population counts at a given location. However, these studies [51,52] have been interested recently in finding out the correlation between the spatiotemporal patterns of crimes and human routine activities, which may ultimately help to provide information about criminals’ movements since the mobility patterns of the general population provide a template for the mobility of criminals [67]. The third application thus investigates the relationship between population dynamics and crime patterns.

Multiple types of data and different spatial units have been proposed to investigate the correlation between human dynamics and crime patterns. The spatial unit of analysis is different according to the format of the data and the official providers. Census units are used in Reference [67] because they are homogeneous in terms of population composition, and Lower Layer Super Output Area (LSOA) units are considered in these studies [13,21,52,66]. Additionally, spatiotemporal characteristics extracted from mobile phone data, such as “the number of times a mobile device communicates with the network”, “timestamp”, “cell ID”, and “the most-contacted tower during daytime or nighttime”, are used in different contexts. For example, the Mobile Phone Origin Destination (MPOD) dataset is used in References [52,66], and the lack of spatial granularity is marked as a weakness, as is the density of signal towers, which is higher in urban areas and lower in suburban ones. This may cause some errors in spatializing the data; thus, a geographical information system (GIS) is used in order to distribute MPOD data across LSOAs.

Statistical models were suggested in the literature to investigate such a correlation. As statistical methods are employed, multiple statistical scores and parameters are used to calculate correlations. For example, in Reference [21], the authors observed that there is no normal distribution; thus, Spearman’s rank correlation coefficient (ρ) statistic was used over Pearson’s product-moment coefficient (r) to calculate correlations between ambient population and crime rates. In Reference [67], the authors applied a discrete choice model to test this hypothesis and determine if the daily mobility flows of the general population can provide a template for the daily mobility of criminals.

The methods used for accomplishing each goal are different, but one aspect is common to all papers: the variables taken into account as ‘crime generators’ are: underground stations; schools (i.e., middle and primary schools); music venues; hotels; hospitals; restaurants; supermarkets; clubs; bars; subway stations; and banks. The mean, standard deviation, minimum, and maximum values were calculated and reported at the census level. For example, there are 11.15 restaurants on average per census unit. In Reference [67], primary schools, hospitals, basic stores, bus stops, supermarkets, banks, and restaurants are listed as ‘crowded spaces’. Song et al. [67] then used the conditional logit model, which aims to analyze the effect of distance, crime generators, and the role population mobility patterns play in offenders’ choice of locations for committing TFP (theft from person). The results showed that all facilities except schools, markets, and bars function as crime generators, and so their presence shows a high likelihood of offenders committing TFP. Furthermore, with larger facilities that have significant effects, such as subway stations, cinemas, or hospitals in the census unit, the odds of being chosen increase by 57.0, 15.4, and 13.5%, respectively. The results also showed that there is a strong correlation between a criminal’s home and crime sites, where criminals often choose places nearby to commit crimes close to where they live.

In the study [21], the variables are residential population, workday population, geo-located Twitter messages, mobile phone activity counts, population 24/7 estimates, and theft from the person who committed the offense. Malleson and Andresen [21] used Getis–Ord Gi*statistics, which examine each location i (LSOAs in this case) together with its neighboring locations j, and then “it calculates whether or not the total number (or rate) of occurrences in i and j is greater or lesser than would be expected by chance when compared to surrounding locations up to a distance from i. If a difference is found, then the areas I and j are assumed to be associated with and different from their surroundings, i.e., a hotspot or coldspot.” The results showed that there is a poor correlation between the residential and ambient populations. On the other hand, strong correlations are noticed between some of the measures of the ambient population (workday population, mobile phone data, and population 24/7 daytime estimates). Moreover, the correlation between thefts and ambient population is stronger than the one between thefts and residential population. Thus, for calculating the crime rate, the ambient population is more suitable than the residential population.

However, Haleem et al. [52] calculated both the ambient and exposed population-at-risk by correlating two datasets: census data to capture residential population counts and mobile phone data to capture transient population counts. This procedure allows for the estimation of the ambient and exposed populations for different time bins. The ambient population-at-risk, thus, was calculated by estimating the residential population at a given spatial unit, summing it with the population entering this unit at a certain period of the day, then subtracting the population existing in this area for the same time of the day. The Spearman’s rank correlation coefficient (ρ as rho) statistic was used to evaluate the correlation between the ambient and exposed population-at-risk measures and violent crimes. The results showed that the exposed population is more significant than the ambient population, and the exposed population measure appears to be a more suitable denominator for exploring violent crimes in public space.

#### 5.1.8. Recent Advances in Method

Recently, a variety of machine learning models and social network analysis techniques have employed mobile phone data [4] to improve criminal network detection, fraud activity detection, and crime prediction.

In recent years, the reconstruction of social networks from mobile phone data by means of graph theory and social network analytics (SNA) has become common in mobile phone data studies. Graph theory refers to the mathematical study of interactions between sets of nodes (otherwise known as vertices) linked by edges. Through the use of social network analysis tools and methods, computer and mobile social networks, including the internet and mobile communications, can be represented graphically in this manner, and graph theory techniques have thus been widely applied in the field of mobile phone data to identify various types of social networks, including the detection of criminal networks [91], the identification of ethnic communities [81], the development of specific socio-economic communities [92], and the determination of geographical networks [93]. This has become possible due to the fact that call data and spatial–temporal data acquired from mobile phones disclose multiple details about a variety of communication links and dynamic networks. The communications recorded on a mobile phone are assumed to constitute a representative part of a person’s overall social networking, with mobile phone data creating a social network among those individuals making or receiving calls or messages, who are classified as actors (nodes) within the network; each link between the actors is then represented by the type of communication (call or message). Empirically, the resulting social networks are constructed based on both the communication behaviors (calling information) and the spatiotemporal information (mobility patterns) extracted from the mobile phone data, allowing observation of a range of social interactions. A network can thus be constructed based on the call patterns created by all the individuals making or receiving calls or messages in the network. A geographical network may, however, be based instead on spatiotemporal information, with the nodes set as geographic locations (e.g., cell towers) and the edges between nodes being represented by the interactions (mobile phone activity) between pairs of these cell towers.

Cavallaro et al. [91] reconstructed the criminal network of the Sicilian Mafia by applying SNA tools and matrices to identify key leaders and their reports, such as bosses and intermediaries. Ficara et al. [94] built a network of suspected criminals based on their calling information; here, the nodes were represented by suspected members and the edges were represented by phone call records. Dileep et al. [95] similarly proposed a forensic detection system to detect the development of suspicious communities based on extracted phone call records.

Statistical methods and machine learning techniques have also been employed to predict crime and detect fraud in other ways. For example, Bogomolov et al. [13] extracted human mobility patterns from mobile phone records to predict crime hotspots in London by using the Random Forest classifier to classify geographical areas into two classes based on whether they displayed high or low crime levels. However, Wu et al. [96] criticized previous data collection methods such as CDRs, Twitter, and Foursquare data in terms of errors in estimating mobility flows for crime prediction, choosing instead to estimate human origin–destination mobility flows using GPS data alongside applied deep learning models such as the gated recurrent units (GRU) model and the graph convolution network (GCN).

While many existing studies have used correlation and regression analysis to investigate the relationship between human dynamics and crime spatial–temporal patterns, Rummens et al. [22] used mobile phone data to investigate whether residential populations or ambient populations have the greatest positive impact on crime rates; the results showed a stronger correlation between ambient populations and crime rates, particularly those for bicycle theft and aggressive theft. Going further, while previous studies examined the impact of ambient and residential population on crime rates, Long et al. [97] aimed to investigate the impact of ambient populations on street robbery rates by applying correlation and regression analysis; they found that the ambient population has a significant effect in terms of reducing opportunistic street robbery and similar crimes. Long and Liu [98] also applied discrete choice models to investigate spatial differences in the patterns of two types of criminals committing street robberies, namely, migrant robbers and native robbers; those results suggested that migrant offenders tend to commit street robberies outside of the old town areas, in industrial areas, while native robbers prefer to commit crimes in villages and older urban areas due to their familiarity with the area, supported by the high mobility of the population and high socioeconomic heterogeneity.

Some studies have employed mobile phone data to detect suspicious and fraudulent behaviors for telecom companies, such as fraud call detection methods based on machine learning. Studies [99,100,101] proposed a range of deep learning models, such as deep neural networks (DNN), convolutional neural networks (CNN), and graph neural networks (GNN), to detect fraudulent phone calls, for example. Using unsupervised learning techniques, such as K-means, density-based spatial clustering of applications with noise (DBSCAN), and hierarchical clustering, References [102,103] also sought to detect fraudulent behaviors for telecom companies, such as fraudulent calls and suspicious call records. Finally, Reference [104] aimed to detect suspicious call behavior by using a range of supervised and unsupervised learning models, including K-means and Random Forest.

### 5.2. RQ2: How Can Identifying Empirical Mobile Phone Data Studies to Predict Human Behavior and Mobility Patterns Contribute to a Clearer Understanding of the Dynamics of Criminal Behavior Contexts through a People- and Place-Centric Perspective?

This question was designed to explore research that has utilized mobile phone data to gain a greater understanding of human behaviors and mobility patterns in urban environments. Experts need to explore people’s actions and activities in the urban areas in which they live and socialize and classify individuals according to their mobility patterns so that the authorities can determine population flows in these zones before and during crimes and provide significant information about criminals’ movements while they are engaging in criminal activities.

These approaches can generate significant information about the tools and methods previously used to analyze mobile phone data, as well as provide a broader understanding of human and/or individual actions and activities.

#### 5.2.1. Human Mobility Patterns in Urban Environments

The spatiotemporal information provided by mobile phone data can help one understand population behavior and mobility patterns in several applications. Investigating population mobility patterns helps one to understand the way humans live, since such patterns reflect the places they visit and stay in the most, as well as their movements during working hours and weekends; thus, many studies use mobile phone data to understand human mobility patterns. To name a few, Thuillier et al. [19] classified individuals into 6 groups based on their daily mobility profiles to comprehend the mobility flows of individuals inside a territory in southwest Paris. These profiles were developed by leveraging the spatiotemporal characteristics extracted from mobile phone data. Ghahramani et al. [71] estimated the frequency of calls at each spatial object (cell towers) to construct a map of hotspots in China. These studies deal with human mobility in urban settings, where crimes are more likely to be committed. Therefore, it is highly important to analyze human mobility patterns inside the city since understanding where and how populations live and socialize and classifying individuals based on their mobility can help to understand population flow [31], which may ultimately help provide information about criminals’ spatial–temporal patterns [67]. This section then reviews important contributions to the study of mobile phone data in urban settings.

#### 5.2.2. Land Use Inference

Long-standing discussions in several disciplines have focused on the connection between land use and human mobility patterns [105] extracted from mobile phone data. This is because understanding the relationship between human activity and land use can help to provide valuable insights into human dynamics and interactions with their physical environment, such as depicting human lifestyles in urban areas and how humans interact and socialize, and investigating the impact of the land use characteristics (commercial, industrial, residential) on urban crime. Thus, many studies have acknowledged the importance of classifying land use to understand the relationship between land use patterns and human activities and interactions.

The classification of land use patterns for visitors or in residential or business areas can be conducted based on extracting human activity characteristics from mobile phone data. Specifically, spatiotemporal and call features extracted from mobile phone data can be used to depict human activity characteristics and infer land use types. For example, References [20,106] explored human activity patterns to infer land use based on spatiotemporal calling volume patterns. Novovic et al. [45] employed user activity variations in space and time to depict human activities; commuting flow patterns to infer land use types was investigated by [107]; and Lenormand et al. [90], along with Ríos and Muñoz [37], inferred land use based on the temporal changes in human activities.

#### 5.2.3. Spatial Distribution of Mobile Phone Presence from Cell Towers to Census Spatial Units

Determining the spatial distribution of mobile phone presence in a cell tower’s coverage area is an important step that needs to be resolved before conducting any further analysis, and it is a common standard procedure for mobile phone data processing. This requires that the spatial configuration of the base stations of a mobile network be matched with the census data. In order to match census data with mobile phone data, we must coincide the spatial scale because the use of different spatial units introduces difficulties when comparing the datasets [3]. Census data are collected according to geographical areas, such as blocks, tracts, or at the country level, whereas mobile phone data are collected at the base station. Thus, the spatial distribution of the base stations should be equal to the spatial units of the census data.

Identifying the position of a mobile device is based on the location of cell towers, which serve as a proxy for the mobile device. The cell towers are represented as Voronoi cells (polygons) using Voronoi tessellation [58]. Voronoi tessellation is used to visualize the position of mobile phones inside the cell towers’ coverage area, which has been approximated as a Voronoi region of a cell tower. The Voronoi diagram contains a point for each cell tower, where the centroid of each point is based on the location of the corresponding cell tower. The resulting Voronoi cells can be viewed as a partition that corresponds to the optimum distribution of towers in a geographical area in a cellular network layout in the real world.

Hence, it is important to perform the spatial distribution of the base stations of a mobile network such that the bases correspond with predefined census units to obtain a fine-scale spatial resolution and to represent the spatial scale of the census data collected at a spatial unit with the mobile phone data collected at the cell towers. This entails the matching of the spatial configuration between the base stations and the census data, which represent the same geographical units.

## 6. Discussion

Previously, this work reviewed the mobile phone data domain and its applications in the areas of crime analysis and urban sensing, developing a consistent taxonomy based on a scientific approach in which studies can be classified at the first level based on human behavior analysis, then subcategorized based on the mobile phone data types used, before being finally classified based on applications derived from each mobile phone data type. This taxonomy helps to answer the research question, shed light on the current state of mobile phone data applications and the current investigation trends in mobile phone data, and highlight existing research gaps. This process was followed by the formulation of two research questions intended to investigate human behavior from both mobility and communication perspectives, the investigation of which helped to generate significant information about the tools and methods previously used to analyze mobile phone data as well as providing a broader understanding of human and individual actions and activities.

The purpose of this section is thus to discuss privacy concerns and investment behavior, and shed light on the emerging common challenges.

### 6.1. Privacy Concerns and Ethical Implications

Previously, this work discussed the benefits that such data can provide for the community and researchers in terms of fighting crime, detecting congestion zones, enhancing urban infrastructure design and urban planning, fighting epidemics, and preventing the spread of infectious diseases. However, mobile phone data are subject to various limitations, including the risk of privacy breaches due to their containing sensitive information about individuals’ locations and their communication information. The potential for a breach of these sensitive details thus raises both privacy concerns and ethical questions about the use of such data.

Various privacy-preserving techniques have been suggested to address this issue. Arcolezi et al. [108] proposed the use of local differential privacy (LDP) techniques, in which each user’s CDRs data are sanitized in the server held by the mobile network operator (MNO) before any data collection processes are performed, while Arfaoui et al. [50] proposed the application of specific anonymization techniques, such as suppression, k-anonymity, and L-diversity, to help guarantee anonymity and prevent personal identification of users. To protect mobile phone users’ location privacy, Gramaglia et al. [109] also proposed a privacy model based on the application of generalization and suppression techniques to achieve k-anonymity in terms of mobile phone spatiotemporal trajectories.

Some authors have also provided recommendations with regard to the multiple ethical implications of such data use. Vespe et al. [110] suggested the development of an expert group of telecommunication engineers, data scientists, lawyers, and data protection and ethics experts, with the aim of addressing various scientific challenges to develop sound data security and protection protocols, alongside the establishment of an Ethical Committee to take on the mission of considering all ethical aspects of work in this field. Similarly, Cinnamon et al. [111] encouraged researchers to facilitate the development of global mobile data usage guidelines, regulations, and standards to provide rapid, secure data access for organizations and researchers that included rapid and efficient techniques for detecting gaps and biases in mobile phone data. Boenig-Liptsin et al. [112] developed a data science lifecycle framework that aims to educate data science students and researchers about the ethical elements of their work and teach or promote ethical principles for responsible data science.

However, privacy implications and ethical concerns still represent challenging obstacles in terms of the use of mobile phone data. In particular, most existing solutions and recommendations have been based on theoretical frameworks rather than empirical work, many of which are impractical and do not conform with national and international data protection regulations. Most existing privacy techniques thus rely heavily on anonymization solutions.

Recently, mobile phone data have been widely used to combat the spread of infectious diseases in emergency situations such as the COVID-19 pandemic and to prevent criminal activities such as terrorist attacks and street robberies. Ignoring the previous advantages of mobile phone data in enhancing quality of life and ensuring citizen safety, some fears and ethical implications have been raised about the violation of people’s privacy and liberty and the imbalance of justice between the right to preserve personal data and law enforcement. However, during emergency situations and natural disasters, government surveillance operations serve to enforce laws against terrorism and serious crime and to place restrictions on people’s basic liberties [113]. For example, in an emergency situation in which suspected terrorists are suspected of criminal activities, the acquisition of their mobile phone data is warranted for forensic analysis and real-time monitoring of their mobility dynamics. Thus, in some cases, it is difficult to strike a balance in data protection during dangerous situations such as terrorist attacks. In addition, creating a balance between controlling national security threats and preserving the personal privacy of suspected phone users is questionable when it comes to the public security and safety of citizens, which are more important than preserving users’ data rights.

Mobile phone data are not the only critical data form that suffers from privacy complications; such concerns have been an ongoing topic with regard to other mobile sensing data. With the growing popularity of mobile wireless devices equipped with various kinds of sensing abilities and a plethora of on-board sensors, the emergence of a large variety of people-centric mobile crowd-sensing (MCS) systems has been rapid [114,115], raising additional concerns. As a result, MCS has become the main emerging sensing paradigm for large-scale sensing applications [116], and it is now used in a range of applications that includes urban dynamics mining, public safety, traffic planning, and environmental monitoring [117].

Mobile crowd-sensing systems are designed to collect city-wide spatiotemporal data [118] from a range of embedded and connected sensors such as GPS sensors, air quality sensors, cardio meters, and health care sensors [119]. However, although these recordings of valuable information offer various benefits for communities in terms of transportation planning and developing public health in communities, such data contain sensitive spatiotemporal information about individuals, such as home addresses, work locations, and health records, which may create possible threats to user privacy if such data are misused or re-identified [120] by attackers.

Privacy-preserving mobile crowd-sensing systems have thus been proposed to preserve and protect user privacy. Agir et al. [121] proposed a form of location privacy protection based on location obfuscation techniques while preserving worker location privacy. Jin et al. [122] designed an auction-based incentive mechanism for MCS systems that enabled data owners to sell location trace information and choose the level of location information to disclose to the MCS system. Chen et al. [123] also proposed a blockchain-based, decentralized framework for MCS systems that aimed to detect fake tasks input by malicious requesters as well as guarantee the task information was not tampered with.

This study contributes to addressing such aspects of privacy concerns in data formats such as MCSs by examining how the approaches proposed can preserve user privacy and protect their information. This is conducted to allow other privacy-preserving mechanisms to be adopted in a mobile phone data context, helping scholars discover new tools and mechanisms for protecting and preserving user privacy.

### 6.2. Investment Behavior

In recent years, advances in artificial intelligence and sensor technology as part of the technological revolution have influenced investment behavior and provided opportunities for corporate development [124,125]. Investments in the field of healthcare have produced highly advanced sensor technology, and many technology companies have invested in digital health products, such as new screening interventions and diagnostic testing. For example, the conversion from the Sanger sequencing method to parallel processing technologies in next-generation sequencing (NGS) has resulted in a significant decrease in the cost of whole-genome sequencing over the past 13 years [126]. There have also been advances in microfluidic technology and devices used to investigate cancer biology and cancer diagnostics. Microfluidic devices are favored for cancer cell detection because of their high sensitivity, control of fluids in the range of micro- to picoliters, and low cost [127]. Similarly, as the high-precision scientific research industry rapidly grows, so do the demands for extremely sophisticated sensor technologies [128]. For example, Reference [129] proposed a photonic spin Hall effect (PSHE) sensor with high sensitivity and the ability to detect both cancer cells and biomedical blood glucose. Thus, with the emergence of health technologies and technological advances in healthcare, individuals are able to make better decisions on how to invest in their health based on the available technology, and firms are able to make better decisions about investing in technology that is both profitable and effective at disease prevention, diagnosis, and cure.

Advances in agricultural technology have also played an important role in farmers’ investment intentions and willingness to invest [130]. Despite the fact that investments in big data analytics solutions are still risky and the cost is substantial [131], firms that invest effectively can benefit from increased customer satisfaction and market performance [132].

### 6.3. Challenges

Although mobile phone data have proven useful in various domains and disciplines with respect to understanding human behavioral patterns, the literature shows a number of serious issues and challenges arising with regard to data access and analysis.

#### 6.3.1. Data Acquisition Challenges

While accessing the required datasets in any study may be hard work, mobile phone data can be among the most difficult to access for several reasons. In particular, mobile phone data at the individual level (CDRs data) contain a wide range of sensitive details about personal characteristics that may expose a person’s identity and personal characteristics. Calabrese et al. [3] thus recommended the use of mobile phone data at the aggregated level and at the cell tower level. However, mobile phone data at the cell tower level lacks calling information, and it is thus not suitable for applications related to human social interactions and communication behaviors. The literature also shows that the use of mobile phone data at the individual level in criminology studies always requires permission from police and law enforcement, which might be a long process.

Several studies have attempted to provide solutions and recommendations to protect and ensure data privacy so as to facilitate access to mobile phone data, such as that conducted by De Montjoye et al. [133], who proposed a remote access model wherein mobile phone data are held by mobile phone operators. However, accessibility remains a significant barrier to using mobile phone data, as governments and businesses are reluctant to make such data public due to privacy concerns [31].

#### 6.3.2. Data Analysis Challenges

The first challenge arising during analysis is that mobile phone data are unlabeled, causing issues around later labeling, especially in supervised ML approaches that require a model to be trained with ground truth data. In the absence of ground truth data, some studies have turned to the use of semi-supervised models in which the model is primed with a small amount of labeled data, such as [20,78], with still others relying on a process of data annotation using domain experts and manual labeling, such as in [39,40,134]; however, the latter requires a lot of manual work.

#### 6.3.3. Challenges Related to the Standardization of Mobile Phone Data Keywords (Terms)

In the literature, we found that there was misunderstanding and misuse of the correct terms for each mobile phone data type. For example, several studies have used various terms or keywords that refer to the mobile phone data, with “the mobile phone data” and “call detailed records data” being the most frequently used in the vast majority of articles. This makes it difficult to search for related papers on this topic. More specifically, when reporting relevant journals and conference proceedings that focus on the topic of mobile phone data in a systematic literature review (SLR), the inconsistent use of keywords by authors makes it infeasible to create search strings that cover all studies within the aforementioned domain. Furthermore, some papers completely omit the relevant “mobile phone data” terms from their keywords or abstracts, necessitating extra time to be dedicated to scanning the full text of such papers, which is a considerably tedious and inefficient job.

## 7. Problem Definition and System Model

This section defines the current problem in mobile phone data with regard to detecting criminal behaviors and proposes a system model to overcome the current challenge.

Problem definition:

The current state of mobile phone data in the context of detecting criminal behaviors and dynamics is still incomplete and inaccurate [4] due to challenges in mobile data pre-processing and analysis.

The problem with pre-processing mobile phone data comes from the fact that raw data can be rough, noisy, and sparse, making it hard to work with. Therefore, the data must be cleaned and preprocessed before being used [71,72,100]. Additionally, during analysis, the use of incomplete or partial mobile phone data, missing values, and partial information (incomplete mobility and calling information) can result in the misleading and inaccurate detection of criminal behaviors and a partial aspect of human behavior [4,79,135,136]. As a result, there is a need to address the issues of incomplete and inaccurate preprocessing and analysis of mobile phone data to improve the detection of criminal behaviors and dynamics.

**Figure 14:**
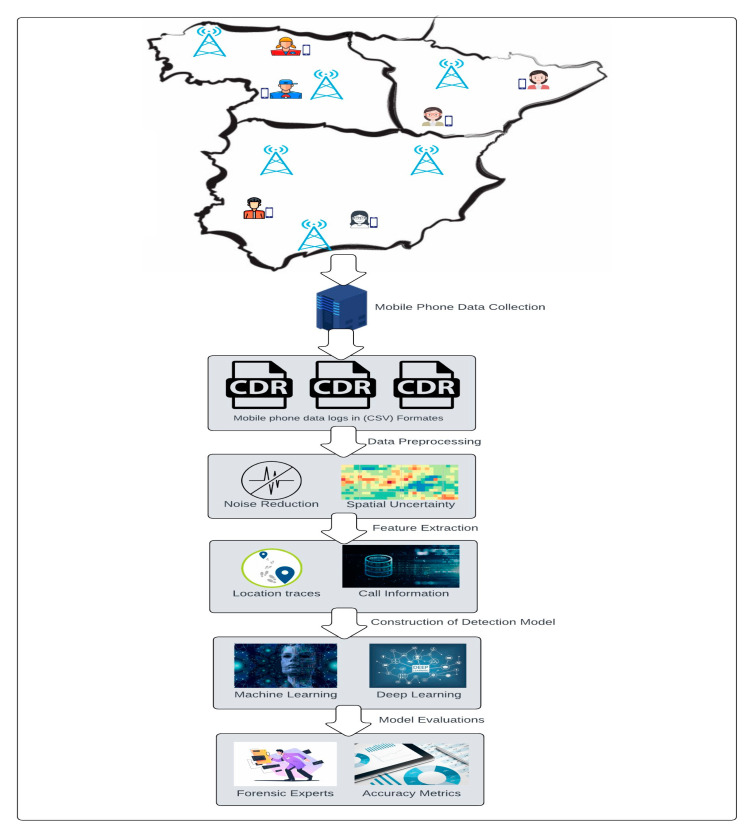
The proposed system model for detecting criminal behaviors.

Proposed model:

Based on a review of the available literature, a forensic analysis system for the detection of criminal behaviors and dynamic activity is proposed for future research. Figure 14 illustrates the different steps of the criminal detection model, which is composed of two stages.

The first stage incorporates mobile phone data at two levels (individual and cell tower levels) to capture different aspects of criminal behaviors (communication behaviors, social networks, and mobility patterns). This stage is then followed by data preprocessing and feature extraction, with several tools and techniques applied, including spatial mapping, feature dimensionality reduction, and uncertainty reduction methods.

The first step in the first stage is the data collection process, which includes the gathering of two types of mobile phone data: mobile phone data at the individual level (CDRs), which can represent the mobility and communication records of suspected criminals, and cell tower location data, which represents a larger sample of the general population, including victims, criminals, suspects, and visitors, with the latter indicating individuals’ locations at the moment a crime takes place based on their use of cell towers in the crime area location. These can thus be used to investigate the relationships between human dynamics and interactions and spatiotemporal crime patterns, along with crime scene data that provides spatiotemporal information on crime incidents according to the official records. The second step is the preprocessing of mobile phone data, which includes the extraction of stay points to detect home location and other meaningful location and spatial mapping techniques to intersect or project mobile network cells into spatial units. The third step involves feature extraction, which helps describe criminal behaviors in terms of spatiotemporal features and call features.

The second stage itself is composed of two steps: analysis and validation. In the analysis step, the construction of a detection model is performed using multiple machine learning classifiers to classify individuals as criminals and non-criminals, thus constructing a classifier that enables the recognition of criminal activities based on spatiotemporal and phone use features. These algorithms are then evaluated to determine the most effective ones, which should yield better results than the other classification algorithms. The results are then used to build up a criminal network of suspected criminals based on applying social network analysis tools and metrics. The construction of a criminal network is conducted to assist law enforcement and crime agencies in identifying the most influential members (who issue commands) and low-level members in a criminal network, clarifying each member’s role in the relevant criminal organizations. The final step is to evaluate the detection model in terms of its accuracy in detecting criminal activity, and this also involves evaluating the model results with the help of forensic experts.

## 8. Future Research Directions, Conclusions, and Limitations

The present review of the existing literature reveals possible directions for future research. The findings highlight aspects that should be considered with regard to data collection, data preprocessing, data analysis, and other considerations.

### 8.1. Data Collection

While collecting mobile phone information, researchers need to consider some points. First, these data should be obtained from leading mobile network operators with a minimum market share of 40–50% and a network providing spatial coverage for 95% of the target population, although these criteria can vary depending on the studies’ goals. Second, researchers need to collect mobile phone data with a full range of users’ attributes, including mobility and communication characteristics. Records that lack all or part of this information hamper analyses and make interpretations of human behavior difficult or even provide misleading evidence.

Last, anonymization is another important step that helps safeguard personal privacy. Before mobile phone data depart storage facilities, mobile telecom operators must anonymize subscribers’ phone numbers and replace them with a unique security identity [5]. During analysis, k-anonymity techniques and approaches should be applied to avoid exposing personal characteristics or leaving enough patterns to reveal individual users’ identities.

### 8.2. Pre-Processing Steps

#### 8.2.1. Labeling Home and Other Meaningful Locations

Previous research has shown that identifying home and other meaningful locations is a crucial step in handling mobile phone data, which is part of pre-processing this information so that further analyses can be conducted using this information, as has been conducted in multiple studies [13,18,34,52,67,137]. Identifying these locations provides a better understanding of human mobility patterns and increases the comprehensiveness of the conclusions that can be drawn from the data. For example, Griffins et al. [12] first established the location of criminals’ homes to clarify their involvement in terrorist attack plots since criminal activities often take place at or near frequently visited locations and criminals often commit crimes close to where they live.

#### 8.2.2. Mapping Population Distribution

The geographical distribution of mobile phone users can be determined based on cell towers’ coverage areas. This crucial step must be completed before proceeding with any further analysis. Defining geographical distribution is, more specifically, a standard technique used in mobile phone data, which has been conducted in multiple studies [57,58,59,60,61,62,63]. It requires a correspondence between census or land cover data and the spatial structure of a mobile network’s base stations because unevenly distributed cell towers will hamper any attempt to map population distribution. Each mobile phone’s geographical location is assigned to a specific cell tower that provides the network signal, so mobile phone location data’s accuracy depends on the towers’ coverage area. The literature shows that researchers often allocate their target population to 1 km- or 500-m-grid cells using methods such as Voronoi tessellation, areal weighting, and dasymetric interpolation.

For instance, Deville et al. [6] applied areal weighted interpolation to the spatial distribution of each cell tower’s coverage area matching a specific spatial unit in order to map the relevant population’s presence at that tower. The spatial unit used can vary between studies and can represent blocks, tracts, administrative units, or any other division that reflects how census data were collected. Deville et al. [6] calculated—for each cell tower *j* simulated and delineated as a Voronoi cell—the population density based on the number of calls or mobile phone presence per cell tower (${\sigma_{c}}_{i}$), in which $c_{i}$ denotes the Voronoi cell associated with cell tower j. Equation (2) was used to estimate mobile phone presence $\sigma_{c_{j}}$ for an area of unit $c_{i}$ that intersects $c_{j}$:

(1) $${\sigma_{c}}_{i}=\frac{1}{A_{c_{i}}}\sum_{c_{j}}\sigma c_{j}\left(c_{i}\cap c_{j}\right)$$

in which ${A_{c}}_{i}$ is spatial unit $c_{i}$’s area and *A*($c_{i}\cap c_{j}$) is the intersection between unit $c_{i}$’s area and Voronoi cell $c_{j}$.

### 8.3. General Recommendations

#### 8.3.1. Recommendations for Improving Interpretation and Justification

Providing a theoretical explanation can play a key role in interpreting differences in results. Justification is absent from the existing literature due to the absence of validation data, so Vanhoof et al. [69] observed that researchers could have trouble discussing results on a theoretical level and determining which outcomes and approaches are better. Blondel et al. [1] also mentioned the need to provide theoretical explanations along with empirical evidence, which can facilitate the interpretation of variations in results and, subsequently, the determination of which findings are significant.

#### 8.3.2. Recommendations for Considering Spatiotemporal Information

Extracting spatiotemporal characteristics to visualize the geographical location of nodes (to visualize the spatial distribution of nodes, or subscribers, in a social network) has been missing in many studies, and current studies rely either on communication information or spatial information to construct social networks. Thus, we recommend including spatiotemporal information with communication information to investigate the interplay between criminal mobility patterns and social interactions. For instance, previous studies [10,11,28] have not considered spatiotemporal information to detect criminals (i.e., the geographic position of nodes is unknown) and have overlooked the spatial position of a node that can connect it to a crime scene or area. Moreover, geographic proximity offers opportunities for face-to-face interactions between individuals. Thus, during graph partitioning into groups of nodes, their geographic locations should be considered to be where nodes have a geographic position. 

In addition, the identification of important members was founded on features extracted from communication information and conducted by placing a weight on the edges between nodes (criminals), such as the maximum number of outgoing phone calls or messages and call duration. Therefore, location data will play an important role in the weighting in that some nodes may not reflect the importance value of a given node in criminal networks. Thus, weighting edges by considering criminals’ mobility patterns could affect results, since the weights of edges reflect their relational strength between the network’s vertices.

Furthermore, few studies have attempted to investigate the relationship and interplay between all aspects of human behavior (mobile communication behavior, social networks, and mobility patterns). This suggestion should arise more in the future for investigating the interplay between communications, social interactions, and mobility patterns through the lens of mobile phone data.

#### 8.3.3. Recommendations to Build a Data-Driven Approach

The study results show that various spatiotemporal and call features have been extracted from mobile phone data to depict or capture criminal behaviors and activities [4]. However, there is no generalized approach in which mobility and social (communication) characteristics can be extracted to capture human behavior, as the literature shows that there are various and multiple spatiotemporal and temporal scales to characterize human and criminal behaviors. Thus, a data-driven framework is needed to determine which measurements and characteristics can be extracted from mobile phone data to visualize and depict different aspects of criminal behaviors, as well as to differentiate and generalize all features and their different functionalities.

#### 8.3.4. Recommendations for Labeling Mobile Phone Data

As mobile phone data are unlabeled, semi-supervised approaches are needed to tackle this issue. A small number of labeled data can be obtained from surveys, censuses, or other geospatial data sources, such as training samples. Mobile phone data can also be labeled by domain experts.

### 8.4. Conclusions and Limitations

This study conducted an SLR to gain comprehensive, up-to-date insights into the current state-of-the-art methods and techniques utilizing mobile phone data in crime-control applications, as well as research that has used mobile phone data to investigate and predict human actions and mobility patterns with reference to urban sensing, which can significantly assist researchers in forming a complete picture of all related crime dimensions. By including studies that have utilized mobile phone data to understand and predict human behavior, the present review made an important contribution to what topics need to be included and discussed to provide a complete understanding of how such studies can help meet objectives in this area. Exploring the movements of human beings in urban areas enables researchers to gain more profound insights into how humans live and the places they most often frequent. The present investigation thus examined the current state of mobile phone data usage in criminology research and shed light on the methods employed to process these data in order to understand the dynamics of human behavior and mobility in the context of urban sensing applications. The latter include estimating and mapping population density, inferring the correlations between human dynamics and land use, and detecting home and work locations.

This study was the first to review the research focused on human mobility and communication behavioral patterns and to make both variables the SLR’s main focus in crime applications, in combination with a lesser emphasis on urban zones. The review covered the most prominent results reported thus far, in particular, analyses of mobile phone data in criminology. The current research is concentrated on detailed data processing and analysis techniques used to understand mobile phone data. The results also include a list of recommendations regarding which techniques and features to use and a discussion of the extant lacunae and obstacles to help researchers and scholars better plan their studies. In addition, the SLR explored which applications have been derived based on human behavioral patterns extracted from mobile phone data.

Although the present research’s approach was based on standard SLR methodology, this study was still subject to limitations. First, the review was intended to provide up-to-date comprehensive coverage of the chosen topic, but the results are neither complete nor should they be regarded as a definitive summary of all the related research. This limitation is primarily due to the exclusion of relevant academic material published in other languages. Nonetheless, the studies included in this review were carefully selected from eight databases and published in international journals. A number of other relevant journals may also have fallen outside the scope of the current review. Those excluded cover, among others, studies of churn prediction, the transportation sector (e.g., transportation planning, transportation mode detection, and commuter trips), anomaly detection, and the epidemiology of infectious diseases, such as COVID-19, in which human mobility patterns have been investigated. These publications were left out because of the large body of literature available and the chosen research objective.

Last, the findings provide unique and potentially useful contributions to the field of criminology, including supporting the conclusion that mobile phone data’s applications in the crime domain still have great potential for further extending the existing knowledge. These approaches can be adopted to explore other domains. On a technical level, the existing analytical perspectives on mobile phone data are somewhat similar in all academic fields that mainly focus on human communication behaviors and mobility patterns. For example, human mobility analyses have been widely conducted in many domains as part of varied practical applications in which mobile phone data facilitated the capture of individuals’ spatial–temporal mobility patterns in a range of human activities associated with urban zones, crime, transportation, and the COVID-19 pandemic. Researchers can adopt more of the techniques and approaches applied in other areas, as well as combine two or more methods, to develop the current understanding of human mobility and interactions further.

In addition, the experiments reported in the mobile phone data literature have often incorporated a variety of different setups and assumptions, each adjusted to complement the techniques applied. In other words, the empirical research conducted with these data has involved various contexts and applications designed to serve each study’s purpose. The current SLR provided a broad overview that can help scholars decide which tools serve their purpose best and discover new uses, thus opening up the possibility of broader—and fewer limitations on—applications so that they can be tailored to serve each study’s specific goals. This finding further justifies this SLR’s consideration of other experiments conducted in different contexts.

## Figures and Tables

**Table 1:** Research questions, explanations, and motivations.

Research Questions	Explanation
RQ1	Several studies have employed mobile phone data to predict crimes and criminal behaviors and to identify criminals and suspects. The present review offers a new and deeper insight into the advanced methods used nowadays in crime applications based on mobile phone data and the benefits of using such data to predict crimes and identify suspects.
RQ2	Mobile phone data have been used in a variety of studies to understand human behaviors and mobility patterns. More precisely, the spatiotemporal information provided by mobile phone data can provide clearer insights into human movements in various applications and academic fields. For instance, mobile phone data has been used to explore human mobility patterns and detect certain types of behaviors in cities and urban zones where criminal activities are much more likely to occur. Mobile phone data have thus been used in different crime and urban sensing applications to serve different purposes, such as defining the actual populations at risk, investigating the relationship between human dynamics and crimes, and inferring land use types based on human dynamics and interactions. For all the above reasons, the defined research question stimulated this investigation of mobile phone data usage in urban sensing, and the results should enable researchers to create more effective methods for extracting useful information from mobile phone data.

**Table 2:** Set of parameters that were applied at the identification phase to filter each database.

Phases in the Selection of Papers	Database	Number of Returned Articles	Timespan	Content Type	Search Within
**Identification Phase**	Scopus (SCP)	n = 753	2014–2022	Article, Review Article	Title, Abstract, and Keywords
	Elsevier ScienceDirect (SD)	n = 183	2014–2022	Article, Review Article	Title, Abstract, and Keywords
	Web of Science (WoS)	n = 588	2014–2022	Article, Review article	Topic (Title, Abstract, and Keywords)
	IEEE Xplore (IEEE)	n = 731	2014–2022	Article, Conference paper	Metadata (Title, Abstract, and keywords)
	SpringerLink (SL)	n = 1371	2014–2022	Article, Conference paper	Abstract
	Multidisciplinary Digital Publishing Institute (MDPI)	n = 62	2014–2022	Article, Review Article	Abstract
	ACM Digital Library (ACM)	n = 83	2014–2022	Article	Title, Abstract
	Science And Geography Education (SAGE)	n = 25	2014–2022	Article	Abstract
**Total**	Papers identified in the identification phase (n = 3796)				

**Table 3:** Eligibility criteria.

Inclusion Criteria (IC)	Exclusion Criteria (EC)
Studies that present novel scientific contributions regarding the use of mobile phone data in detecting and identifying suspects and criminals.	Articles using mobile phone data in the context of smart marketing; the transportation sector (such as transportation planning, transport mode detection, and traffic prediction); economic forecasting; and health sciences research.
Studies that incorporate mobile phone data to predict crimes, perform spatial–temporal crime analysis, or have any other bearing on criminological research.	Studies using mobile phone data to measure human mobility in relation to the epidemiology of infectious diseases.
Studies that investigate the use of mobile phone data in home and work location detection; mapping human population density; classifying land use types; **detecting social interaction networks**; and others.	Publications that are not written in the English language.

**Table 4:** Top 10 most cited articles and reviews.

Reference	Domain/application	Citation Count	Year
[6]	Mapping human population density	786	2014
[1]	Constructing social networks from mobile phone data	574	2015
[34]	Detecting cities’ hotspots	397	2014
[20]	Classifying urban land uses	347	2014
[13]	Predicting crime	325	2014
[3]	Developing urban sensing applications based on mobile phone data	306	2014
[35]	Inferring home and work locations	292	2014
[36]	Mapping society-wide interaction networks of two European countries	268	2014
[10]	Detecting criminal networks	181	2014
[21]	Investigating correlations between human mobility patterns and crime rates (i.e., crime statistics)	112	2016

**Table 5:** Prior research on suspect identification.

Reference	Features	Description
[82]	Spatiotemporal features	This study aimed to identify the most probable suspects in a given case by correlating CDRs with other data sources, such as digital video recorders (DVRs) and base transceiver station (BTS) log files to help investigators with otherwise insufficient evidence pinpoint hidden details about their suspects and gather further digital evidence to show how a crime is committed. The author extracted spatiotemporal information, such as the suspects’ various trajectories, from CDR data and cell tower IDs that showed each suspect’s home cell tower location along with other visited cell tower locations, to prove involvement in the crime.
[83]	Call features	This study aimed to identify suspects based on their calling characteristics, including any phone calls made or received by the suspects at the crime scene, in conjunction with archived CDRs data drawn from a central database that contained details on previously convicted criminals whose names had been recorded in older cases.
[84,85]	Call and spatiotemporal features	These studies aimed to improve the identification performance of suspects in terms of efficiency, effort, and scalability. To achieve this, they proposed a system-based big data analytic process to extract communication and mobility information from CDRs data, including aspects such as the most frequent caller, the number of times the suspect called other suspects, call frequency, suspect trajectories, and the most visited location based on the most frequently used cell tower.
[86]	Spatiotemporal features	This study proposed a terrorist detection system that aims to detect suspicious activities based on user trajectories.
[87]	Call and spatiotemporal features	This study aimed to investigate additional details by identifying suspects and their accomplices. To achieve this, the authors extracted calling and spatiotemporal information from the CDRs, such as calls made and received by suspects and suspects’ trajectories near crime locations, then applied MariaDB, an open-source relational database management system (RDBMS), to analyze the CDRs data.
[9,47]	Call features	Rather than applying traditional methods, these studies proposed machine learning methods to tackle the identification process. They applied classification algorithms that aimed to separate suspects from non-suspects based on communication behaviors.
[88]	Call features	Going one step further, some studies discussed the challenges associated with analyzing CDRs to identify suspects. Marshall and Miller [88] aimed to present different techniques and scenarios suspects might use to avoid recording of their communication and mobility activities, such as stealth SIM, voice changing, roaming callback, and call obfuscation.

**Table 6:** List of methods, analysis approaches, and metrics in crime applications.

Reference	Analysis Perspective	Analysis Approach	Algorithm/Measure	Network Metrics/ Parameter	Limitation
[28]	Communication behaviors	SNA	Detection algorithm (Prim’s Minimum Spanning Tree Algorithm)	Edge-centric	Missing location data, greedy algorithm
[10]	Communication behaviors	SNA	Detection algorithm (Girvan–Newman and Fruchterman–Reingold)	Edge-betweenness centrality	Complex network, detection only based on communication information, greedy algorithm
[11]	Communication behaviors	SNA	The concept space approach (space algorithm)	Vertex-centric	Suitable for small networks, detection only based on communication information
[51]	Mobility patterns	Regression and Correlation Analysis	Akaike information criterion (AIC), spatial autocorrelation (SA) using Pearson’s Correlation, and negative binomial regression model (NBM)	Offender anchor points.	Detection only based on spatiotemporal information
[12]	Mobility patterns	Statistical and Correlation Analysis	Spearman’s rank coefficient (ρ) statistics, Pearson’s correlations, and the cumulative distribution function	Offender anchor points.	Detection only based on spatiotemporal information

## Data Availability

Not applicable.
